# Relative contributions of family history and a polygenic risk score on COPD and related outcomes: COPDGene and ECLIPSE studies

**DOI:** 10.1136/bmjresp-2020-000755

**Published:** 2020-11-25

**Authors:** Matthew Moll, Sharon M. Lutz, Auyon J. Ghosh, Phuwanat Sakornsakolpat, Craig P. Hersh, Terri H. Beaty, Frank Dudbridge, Martin D. Tobin, Murray A. Mittleman, Edwin K. Silverman, Brian D. Hobbs, Michael H. Cho

**Affiliations:** 1 Channing Division of Network Medicine, Department of Medicine, Brigham and Women's Hospital, Boston, Massachusetts, USA; 2 Division of Pulmonary and Critical Care Medicine, Brigham and Women’s Hospital, Boston, Massachusetts, USA; 3 Harvard Medical School, Boston, Massachusetts, USA; 4 PRecisiOn Medicine Translational Research (PROMoTeR) Center, Department of Population Medicine, Harvard Medical School, Boston, Massachusetts, USA; 5 Harvard Pilgrim Health Care, Wellesley, Massachusetts, USA; 6 Department of Medicine, Mahidol University Faculty of Medicine Siriraj Hospital, Bangkok, Bangkok, Thailand; 7 Johns Hopkins University Bloomberg School of Public Health, Baltimore, Maryland, USA; 8 Health Sciences, University of Leicester, Leicester, Leicestershire, UK; 9 Genetic Epidemiology Group, Department of Health Sciences, University of Leicester, Leicester, Leicestershire, UK; 10 National Institute for Health Research Leicester Respiratory Biomedical Research Centre, Glenfield Hospital, Leicester, UK; 11 Epidemiology, Harvard University T H Chan School of Public Health, Boston, Massachusetts, USA; 12 Department of Medicine, Beth Israel Deaconess Medical Center, Boston, MA, United States

**Keywords:** COPD epidemiology, emphysema, tobacco and the lung

## Abstract

**Introduction:**

Family history is a risk factor for chronic obstructive pulmonary disease (COPD). We previously developed a COPD risk score from genome-wide genetic markers (Polygenic Risk Score, PRS). Whether the PRS and family history provide complementary or redundant information for predicting COPD and related outcomes is unknown.

**Methods:**

We assessed the predictive capacity of family history and PRS on COPD and COPD-related outcomes in non-Hispanic white (NHW) and African American (AA) subjects from COPDGene and ECLIPSE studies. We also performed interaction and mediation analyses.

**Results:**

In COPDGene, family history and PRS were significantly associated with COPD in a single model (P_FamHx_ <0.0001; P_PRS_<0.0001). Similar trends were seen in ECLIPSE. The area under the receiver operator characteristic curve for a model containing family history and PRS was significantly higher than a model with PRS (p=0.00035) in NHWs and a model with family history (p<0.0001) alone in NHWs and AAs. Both family history and PRS were significantly associated with measures of quantitative emphysema and airway thickness. There was a weakly positive interaction between family history and the PRS under the additive, but not multiplicative scale in NHWs (relative excess risk due to interaction=0.48, p=0.04). Mediation analyses found that a significant proportion of the effect of family history on COPD was mediated through PRS in NHWs (16.5%, 95% CI 9.4% to 24.3%), but not AAs.

**Conclusion:**

Family history and the PRS provide complementary information for predicting COPD and related outcomes. Future studies can address the impact of obtaining both measures in clinical practice.

**Key messages**

- We sought to understand whether a Polygenic Risk Score (PRS) for chronic obstructive pulmonary disease (COPD) provides complementary or redundant information compared with family history for predicting COPD and related outcomes.
- Family history and the PRS provide complementary information for predicting COPD and related phenotypes, though the PRS appears to be an overall stronger predictor for COPD-related phenotypes than family history alone; in mediation analysis, only 16.5% of the effect of family history on COPD was mediated by the Polygenic Risk Score in non-Hispanic whites, and less in African Americans.
- Both family history and the Polygenic Risk Score are needed to understand and individual’s risk for COPD.

## Introduction

Chronic obstructive pulmonary disease (COPD) is characterised by fixed airway obstruction, and is a leading cause of morbidity and mortality worldwide.^1^ This disease primarily develops in the context of cigarette smoking or biomass fuel exposure. However, only a minority of smokers develop this disease.^2 3^ Certain individuals may have increased genetic susceptibility to developing COPD, and studies have estimated the proportion of COPD liability variance explained by genetic factors (ie, heritability) to be approximately 40%.^4–6^

Before the advent of molecular genotyping, family history was the main method to assess a familial contribution to risk of complex diseases (ie, where both genetic and environmental factors influence risk). Family history is a composite measure of common and rare variants as well as shared environmental risk factors.^7^ Family history is a known risk factor for COPD, with a population attributable risk of ~17%.^8^ Despite the clinical use of family history, there are several limitations to using family history as a risk factor for a complex disease. First, a report of family history depends on the disease occurring and being diagnosed in relatives (typically first degree relatives), and does not capture those at high risk for the disease who may have not yet developed severe disease or received a diagnosis.^7^ Second, those affected by disease often do not have any known family history of the disease.^9^ Third, family history is subject to recall bias and often is unknown or incorrect. Finally, family history can reflect shared environmental exposures as well as genetics.

Genome-wide association studies (GWASs) have identified numerous single nucleotide polymorphisms (SNPs) associated with low lung function levels and COPD,^10–14^ though these variants typically exert a small effect on disease risk and account for a small proportion of the phenotypic variability. Pooling thousands to millions of genetic variants of small effect into a composite genetic risk score can improve prediction.^10 13 14^ We recently developed a Polygenic Risk Score (PRS) highly predictive for COPD, multiple CT imaging phenotypes, and patterns of lung growth and decline that are thought to be important in disease pathogenesis. The PRS added information to traditional risk factors (age, sex, pack-years of smoking), and increased predictive power for COPD.^15^

The relationship between genetic risk scores and family history in predicting COPD is unclear. Recently, studies have assessed relative contributions of genetic or polygenic risk scores and family history for other complex diseases. Genetic risk scores improved prediction of incident coronary heart disease, and were reported to be independent of family history.^16 17^ In schizophrenia, a PRS and family history were found to provide complementary information; however, the PRS was found to interact with family history, and 17.4% of the effect of family history on schizophrenia was mediated through the PRS.^18^ These data suggest that family history and PRS can provide complementary information in complex disease, and the way family history interacts with or is mediated by genetic risk may vary among diseases.

We hypothesised that PRS and family history would provide complementary information for predicting COPD and related phenotypes. To test this hypothesis, we assessed the predictive performance of the PRS and family history. We additionally asked whether the relative impact of family history and PRS differed among these related phenotypes; whether PRS and family history interact on the multiplicative or additive scale. Finally, we sought to identify causal relationships between family history and the PRS using mediation analysis.

## Methods

### Study populations

All participants gave informed consent (see online supplemental for further details).

10.1136/bmjresp-2020-000755.supp1 Supplementary data

### COPDGene

We included participants from the COPDGene study, which has been previously described.^19^ Briefly, this study included 10 192 non-Hispanic white (NHW) and African American (AA) participants aged 45–80 years with ≥10 pack-years of smoking. COPDGene was initially designed as a case–control study,^19^ and was later extended to a longitudinal study. Only baseline data were included in the current analyses.

### ECLIPSE

The ECLIPSE study was a 3-year longitudinal study to identify surrogate endpoints in 2746 participants (2164 COPD cases) associated with disease progression and exacerbations in COPD.^20^ Only baseline data were included in the current analyses. Participants were white, 40–75 years of age at enrollment, and had ≥10 pack-years of smoking history.

**Figure 1:**
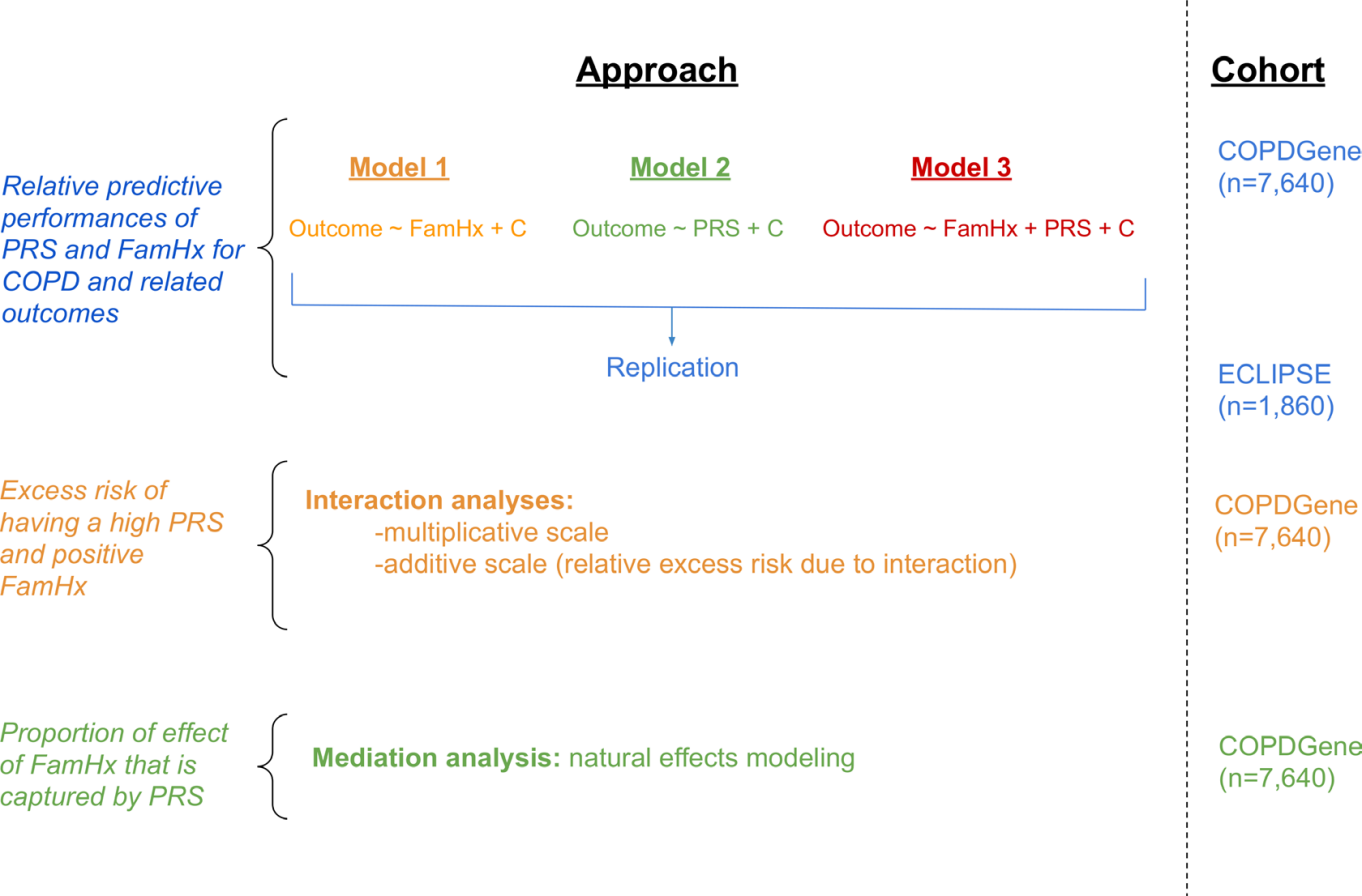
Schematic of study design. COPD, chronic obstructive pulmonary disease; PRS, Polygenic Risk Score. FamHx = family history of COPD. PRS = polygenic risk score. C = covariates.

In both cohorts, baseline demographic, spirometry, chest CT imaging, family history, exacerbation frequency over the last 12 months, and number of severe exacerbations (requiring emergency room visits or hospitalisations) were recorded. A schematic of the study design is shown in figure 1.

### Patient and public involvement

Patients or the public were not involved in the design, or conduct, or reporting, or dissemination plans of our research.

### Statistical analyses

#### Outcomes

We used COPDGene as our discovery cohort and ECLIPSE as a replication cohort. The primary outcome was moderate-to-severe COPD, defined as postbronchodilator forced expiratory volume in 1 s (FEV_1_) <80% predicted and FEV_1_/forced vital capacity (FVC) <0.7. Control subjects had FEV_1_ ≥80% predicted and FEV_1_/FVC ≥0.7; global initiative for chronic obstructive lung disease^1^ (GOLD) 1 and preserved ratio with impaired spirometry subjects were excluded. Secondary outcomes included frequent exacerbations (>1 exacerbation in prior 12 months), severe exacerbations (exacerbation requiring hospitalisation or an emergency room visit), death, baseline 6 min walk distance (6MWD), the body mass index, obstruction, dyspnoea, exercise capacity (BODE index),^21^ total St. George’s Respiratory Questionnaire score and CT imaging phenotypes (percent emphysema determined by the percent low attenuation area of the lungs ≤950 Hounsfield units (≤950 HU],^22^ the HU value at the 15th percentile of the lung density histogram on inspiratory scans (Perc15),^23^ square root of wall area (WA) of a hypothetical airway with an internal perimeter of 10 mm (Pi10),^24^ mean WA percent (WA%).^22^ Additional outcomes only available in COPDGene included visual CT subtypes of destructive emphysema and airway pathology.^25^ We adjusted for multiple comparisons using the Bonferroni method.

### Predictors

Consistent with prior analyses in the COPDGene study, we defined a family history of COPD by self-reported maternal or paternal history of COPD, chronic bronchitis or emphysema.^8^ All participants responded to the questionnaire; we considered a response of ‘unknown’ as a ‘no’ response. In the ECLIPSE study, there was not a question about family history of COPD, so a maternal or paternal family history of chronic bronchitis or emphysema was considered a positive family history for COPD. In ECLIPSE, there were no missing or ‘unknown’ responses. For sensitivity analyses, we repeated the analysis of the primary outcome (1) excluding subjects that reported not knowing their family history of COPD, and (2) using a harmonised definition of family history in COPDGene to match ECLIPSE (ie, maternal or paternal family history of emphysema or chronic bronchitis). Given the imbalance of cases and controls in ECLIPSE, we repeated the main analyses using Firth regression to adjust for potential bias.

Development of the PRS was previously described.^15^ Briefly, the PRS was derived from GWASs of FEV_1_ and FEV_1_/FVC in UK Biobank and SpiroMeta consortium participants.^13^ For each individual, we calculated a PRS for FEV_1_, a separate PRS for FEV_1_/FVC, then created a combined PRS using a weighted sum of these two scores. For the primary analysis, we treated the PRS as a continuous variable. The association of the continuous PRS with each outcome was reported with respect to 1 SD increase in the PRS. We also dichotomised the PRS (1=top tertile; 0=bottom two tertiles) to allow comparison of ORs for the PRS with ORs for family history; tertiles were chosen as about one-third of controls also had a positive family history, so this dichotomisation allowed comparison of the PRS to family history. The PRS was treated as a continuous variable for interaction and mediation analyses. We performed biserial correlation between family history and the PRS assuming 10 bins for the PRS using the polycor R package.^26^

### Model specifications and performance evaluations

For both family history and the PRS (dichotomised), we calculated an adjusted OR, attributable fraction in the exposed (AF_exposed_), and attributable fraction in the population (AF_population_) with respect to the primary outcome as detailed in.^27^ We built three regression models (linear or logistic, as appropriate) for each outcome: model (1) Outcome ~family history +covariates; model (2) Outcome ~PRS+ covariates and model (3) Outcome ~family history+PRS+covariates. All models were adjusted for age, sex and pack-years of cigarette smoking. When frequent or severe exacerbations was the outcome, the model was additionally adjusted for baseline FEV_1_% predicted and current smoking as these are known predictors of COPD exacerbations. For baseline 6 MWD, models were adjusted for height and weight. For death as the outcome, models were adjusted for the BODE index, which is a strong predictor of mortality in COPD.^21^ For all CT imaging outcomes, the models were adjusted for CT scanner.

We calculated the area under the receiver operator characteristic curve (AUROC) and scaled Brier scores^28 29^ for logistic regression models to assess predictive performance of each model. AUC and 95% CIs were calculated with the pROC R package.^30^ For continuous outcomes, we calculated adjusted R^2^ and mean squared error (MSE) values to compare models.

### Meta-analyses

To estimate effects including all subjects, we meta-analysed the results of models from COPDGene and ECLIPSE that included both PRS and family history (model 3) using the meta R package.^31^ Fixed effects were reported as there was little study heterogeneity when comparing the effects of family history on COPD (I^2^=0). We constructed boxplots of ORs, betas and 95% CIs, as appropriate.

### Interaction analyses

For the primary outcome, moderate-to-severe COPD (hereafter, referred to as ‘COPD’), we performed an interaction analysis in the COPDGene study. We tested for a multiplicative interaction using a logistic regression model including family history, PRS, and a family history by PRS interaction term. We also evaluated the joint effects of family history and the dichotomised PRS, and performed stratified analyses. We tested for an additive interaction by calculating the relative excess risk due to interaction (RERI).^27 32 33^

### Mediation analyses

To test for mediated effects, we evaluated whether the COPD association with family history is mediated through the PRS by testing for the natural direct/indirect effect (NDE/NIE)^34–36^ using Medflex^37^ in R. Interaction and mediation models were adjusted for age, sex and pack-years of cigarette smoking.

All analyses were done in R V.3.6.0 (www.r-project.org). Normality for continuous variables was assessed by visual inspection of histograms and Shapiro-Wilk tests. Results are shown as mean±SD or median (IQR), as appropriate. Differences in continuous variables were assessed with Student’s t-tests or Wilcoxon tests. Categorical variables were compared by analysis of variance or Kruskal-Wallis tests, as appropriate.

## Results

### Characteristics of study populations

Characteristics of the study populations are shown in table 1. We included 5174 NHW and 2466 AA participants from the COPDGene study. COPD cases tended to be older, had greater mean pack-years of cigarette smoking, higher exacerbation rates, higher mean PRS and a greater percentage of individuals reporting a family history of COPD.

**Table 1:** Characteristics of cohorts

Characteristic	COPDGene NHW		COPDGene AA		ECLIPSE	
	Controls	Cases	Controls	Cases	Controls	Cases
n	2506	2668	1713	753	147	1713
Age in years (mean (SD))	59.54 (8.71)	64.73 (8.13)	52.98 (6.17)	59.31 (8.15)	57.32 (9.55)	63.64 (7.10)
Sex (no female, (%))	1283 (51.2)	1187 (44.5)	704 (41.1)	341 (45.3)	63 (42.9)	563 (32.9)
BMI (kg/m^2^) (mean (SD))	28.83 (5.67)	27.98 (6.02)	29.10 (6.22)	27.89 (6.87)	27.34 (4.17)	26.53 (5.54)
Current smoking (no (%))	1016 (40.5)	912 (34.2)	1492 (87.1)	445 (59.1)	46 (37.7)	475 (35.4)
Pack-years cigarette smoking (mean (SD))	38.78 (21.15)	56.09 (27.27)	36.58 (19.68)	42.82 (23.33)	31.01 (25.94)	50.50 (27.47)
SGRQ total score (mean (SD)) (mean (SD))	17.05 (16.97)	40.79 (20.86)	23.78 (20.17)	44.79 (22.82)	9.45 (13.11)	50.91 (19.89)
6 min walk distance (ft) (mean (SD))	1558.32 (330.46)	1207.83 (388.93)	1365.12 (351.65)	1042.20 (395.39)	NaN (NA)	1084.17 (355.24)
BODE (mean (SD))	0.44 (0.93)	3.39 (2.45)	0.96 (1.26)	3.78 (2.49)	NaN (NA)	3.26 (2.13)
Frequent exacerbations (no (%) >1 per year)	45 (2.5)	214 (13.9)	36 (3.9)	56 (14.5)	0 (0.0)	775 (45.7)
Severe exacerbations (no (%))	63 (3.5)	295 (19.2)	69 (7.6)	107 (27.6)	0 (0.0)	542 (31.6)
FEV1 % predicted (mean (SD))	94.85 (13.07)	48.84 (17.80)	96.57 (14.33)	51.42 (17.85)	108.87 (12.28)	47.11 (15.47)
FEV1/FVC ratio (mean (SD))	0.77 (0.06)	0.48 (0.13)	0.79 (0.06)	0.52 (0.12)	0.80 (0.05)	0.44 (0.11)
Combined FEV1 and FEV1/FVC PRS (mean (SD)) (mean (SD))	−0.27 (0.97)	0.25 (0.96)	−0.09 (0.99)	0.20 (1.00)	−0.60 (0.98)	0.05 (0.98)
Family history of COPD, chronic bronchitis or emphysema	710 (28.3)	991 (37.1)	247 (14.4)	145 (19.3)	51 (34.7)	717 (41.9)

Those with frequent exacerbations had more than one exacerbation per year requiring steroids and/or antibiotics. Severe exacerbations including worsening in respiratory health requiring emergency room visit or hospitalisation.

AA, African American; BMI, body mass index; BODE, body mass index, obstruction, dyspnoea, exercise capacity; COPD, chronic obstructive pulmonary disease; FEV1, forced expiratory volume in 1 s; FVC, forced vital capacity; NHW, non-Hispanic white; PRS, Polygenic Risk Score; SGRQ, St. George’s Respiratory Questionnaire.

In ECLIPSE, we included 1860 European-ancestry participants, the majority of which were COPD cases (147 controls, 1713 COPD cases). Differences between cases and controls were similar to those observed in the COPDGene sample, with the exception of current smoking status; COPDGene cases had a lower proportion of current smokers compared with controls, while ECLIPSE cases and controls had similar rates of current smoking. 6MWD, and therefore BODE, were not available in ECLIPSE controls.

### PRS and family history are complementary for predicting COPD

The distribution of PRS values in those with (NHW: n=1701, AA: n=392) and without (NHW: n=3473, AA: 2074) a family history of COPD in the COPDGene study is shown in online supplemental figure S1. Visual inspection reveals a small difference in the distribution of PRS values, which was statistically significant in NHWs (t-test p value=2e-7; biserial correlation coefficient=0.093, p=0.0062), but not AAs (t-test p=0.47; biserial correlation coefficient=0.022, p=0.64).

In each cohort, we considered three logistic regression models for moderate-to-severe COPD (see Methods, figure 1). The resulting ORs for variables included in each model are shown in table 2, and unadjusted ORs are in online supplemental table S1. When modelled separately, holding all other covariates constant, family history was associated with a 1.77 OR for COPD (95% CI 1.6 to 2.0, p=4.3e-17) in COPDGene NHW and 1.71 OR for COPD (95% CI 1.4 to 2.2, p=9.5e-6) in COPDGene AA participants. The PRS was associated with a 2.13 OR for COPD (95% CI 1.98 to 2.28, p=9.0e-95) per SD increment in the risk score in COPDGene NHW and 1.50 OR for COPD (95% CI 1.4 to 1.6, p=2.5e-17) per SD increment in the risk score in COPDGene AA participants. When modelled together, both family history and the PRS were associated with COPD (COPDGene NHW: Family history: OR=1.67 (95% CI 1.45 to 1.92, p=1.6e-12); PRS: OR=2.11 (95% CI 1.97 to 2.27, p=5.0e-92); COPDGene AA: Family history: OR=1.74 (95% CI 1.4 to 2.2, p=8.8e-6); PRS: OR=1.5 (95% CI 1.4 to 1.7, p=3.2e-17)). In the ECLIPSE sample; when modelled alone (model 1) family history was not significantly associated with risk of COPD, though when modelled along with PRS (model 3), family history was significantly associated with risk of COPD (table 2). Due to the imbalance of cases and controls in ECLIPSE, we repeated the analysis using Firth regression for ECLIPSE, and observed similar results. Similar results were also found when dichotomising the PRS (online supplemental table S2). Excluding subjects who reported not knowing their family history of COPD, we found consistent results (online supplemental tables S3 and S4). As an additional sensitivity analysis, we harmonised the definitions of family history of COPD such that the definition in COPDGene matched that in ECLIPSE; we again observed consistent results (online supplemental table S5).

**Table 2:** Associations of family history and PRS in three logistic regression models of moderate-to-severe COPD: model 1 (COPD~Family history+age+pack years+sex); model 2 (COPD ~ PRS+age+pack years+sex+principal components of genetic ancestry); model 3 (COPD ~ family history+PRS+age+pack years+sex+principal components of genetic ancestry)

Variable	Model 1		Model 2		Model 3	
	OR (95% CI)	P value	OR (95% CI)	P value	OR (95% CI)	P value
	**COPDGene Non-Hispanic white**					
Family history	1.77 (1.55 to 2.03)	4.30E-17	NA	NA	1.67 (1.45 to 1.92)	1.60E-12
PRS	NA	NA	2.13 (1.98 to 2.28)	9.00E-95	2.11 (1.97 to 2.27)	5.00E-92
	**COPDGene African-American**					
Family history	1.71 (1.35 to 2.17)	9.50E-06	NA	NA	1.74 (1.36 to 2.21)	8.80E-06
PRS	NA	NA	1.5 (1.36 to 1.64)	2.50E-17	1.5 (1.36 to 1.65)	3.20E-17
	**ECLIPSE**					
Family history	1.33 (0.91 to 1.94)	0.14	NA	NA	1.69 (1.13 to 2.53)	0.011
PRS	NA	NA	2.02 (1.65 to 2.47)	6.70E-12	2.01 (1.64 to 2.45)	8.70E-12

Bonferroni-adjusted level of significance is 0.05/3 models=0.017. The PRS was treated as a continuous variable, and ORs are associated with 1 SD increase in the PRS.

AA, African American; COPD, chronic obstructive pulmonary disease; NA, not applicable; NHW, non-Hispanic white; PRS, Polygenic Risk Score.

We calculated the attributable fraction in the exposed (AF_exposed_) and in the population (AF_population_) for both family history and the dichotomised PRS (online supplemental table S6). In COPDGene NHW participants, the AF_exposed_ for family history was 0.4 and for the dichotomised PRS was 0.7. The AF_population_ for family history was 0.15, and for the PRS was 0.29. In COPDGene AA participants, the AF_exposed_ for family history was 0.42 and for the dichotomised PRS was 0.45; the AF_population_ for family history was 0.087, and for the PRS was 0.18.

### PRS and family history are complementary for predicting COPD and COPD-related outcomes

**Figure 2:**
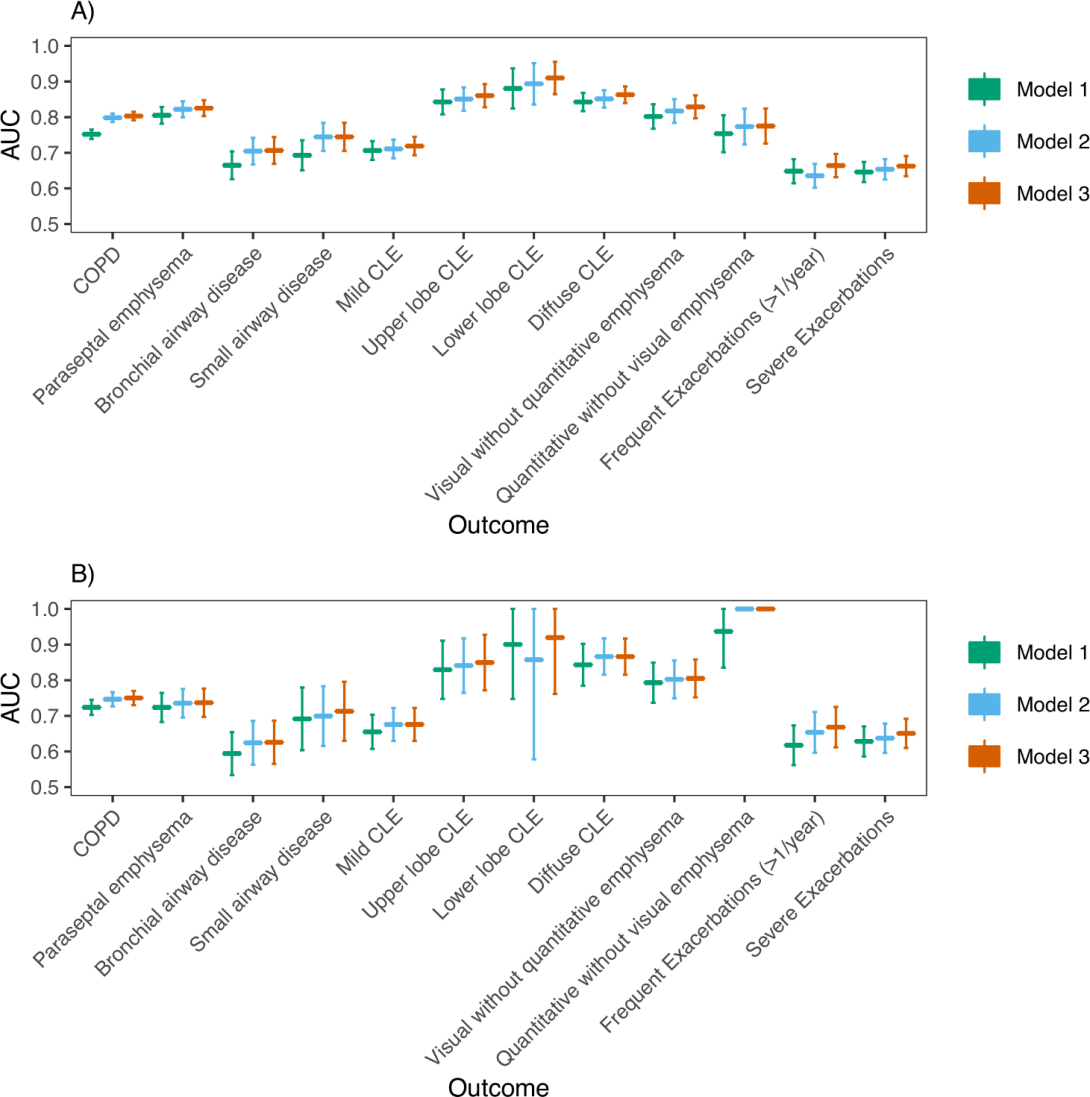
(A) AUC analysis in COPDGene NHWs: Predictive performance (AUC) of three logistic regression models for the discrimination of outcomes shown on the x-axis. The PRS was analysed as a continuous variable. For each outcome, three models were trained: model 1 (outcome ~ family history + age + sex + pack-years), model 2 (outcome ~ PRS + age + sex + pack-years + principal components of genetic ancestry) and model 3 (outcome ~ family history + PRS + age + sex + pack-years + principal components of genetic ancestry). B) AUC analysis in COPDGene AAs. Abbreviations are listed in the caption for table 3. P values comparing model AUCs are shown in online supplemental table S8 and were considered significant if less than Bonferroni-corrected level of significance (p<0.05/12=0.0036). AA, African American; AUCs, area under the curve; CLE, centrilobular emphysema; PRS, Polygenic Risk Score.

We also considered three analogous models for a range of COPD-related outcomes (table 3 and online supplemental table S7). In COPDGene NHW participants (figure 2; online supplemental figures S2 and S3), AUCs for discriminating COPD affection status from family history (model 1), PRS (model 2) and the combined predictors (model 3) were 0.752 (95% CI 0.739 to 0.765), 0.798 (95% CI 0.786 to 0.810), 0.803 (95% CI 0.791 to 0.815), respectively. In COPDGene AA participants, AUCs for discriminating COPD affection status from family history (model 1), PRS (model 2), and the combined predictors (model 3) were 0.724 (95% CI 0.703 to 0.745), 0.747 (95% CI 0.726 to 0.767) and 0.750 (95% CI 0.730 to 0.770), respectively. The AUC for a model containing both family history and the PRS was statistically significantly higher than models with only PRS in NHWs (p=0.00035), but not AAs (p=0.1), and was statistically significantly higher than models with only family history in both NHWs and AAs (p (NHW)=6.1e-29, p (AA)=1.0e-6). P values comparing AUCs for models trained in all cohorts are shown in online supplemental table S8). As an additional performance measure, we also calculated scaled Brier scores (online supplemental table S9), which suggest similar trends in predictive performance between models. For quantitative outcomes, we found models including both family history and PRS gave a better fit compared with those with family history alone, and often PRS alone, as evidenced by a higher adjusted R^2^ and lower MSE (online supplemental table S10). Family history was associated with frequent exacerbations (≥2 exacerbations per year) in COPDGene NHWs, and the PRS, but not family history, was significantly associated with a higher Pi10. Otherwise, both family history and the PRS were concordantly associated even though some were not statistically significant.

**Table 3:** Association of family history and PRS with outcomes (left)

Outcome	Covariates	COPDGene NHW				COPDGene AA				ECLIPSE			
		Family history (OR or beta (95% CI))	P value	PRS (OR or beta (95% CI))	P value	Family history (OR or beta (95% CI))	P value	PRS (OR or beta (95% CI))	P value	Family history (OR or beta (95% CI))	P value	PRS (OR or beta (95% CI))	P value
6 min walk distance	Height, weight	−27 (−49 to −5.4)	0.016	−33 (−43 to −23)	1.40E-10	−36 (−75 to 3.2)	0.076	−17 (−32 to −2.5)	0.019	−29 (−66 to 8.2)	0.14	2.1 (−16 to 20)	0.82
SGRQ Total Score		0.27 (0.21 to 0.33)	1.20E-16	0.14 (0.11 to 0.17)	3.00E-21	0.39 (0.26 to 0.52)	5.80E-09	0.061 (0.014 to 0.11)	0.011	3.6 (1.4 to 5.8)	0.00084	2.2 (1.2 to 3.2)	2.70E-05
Frequent Exacerbations	FEV1 % predicted, current smoking	1.65 (1.26 to 2.16)	0.00031	0.91 (0.79 to 1.04)	0.18	1.39 (0.83 to 2.34)	0.21	1.2 (0.96 to 1.5)	0.11	1.2 (0.94 to 1.52)	0.15	0.99 (0.88 to 1.11)	0.83
Severe Exacerbations	FEV1 % predicted, current smoking	1.17 (0.91 to 1.49)	0.22	0.97 (0.86 to 1.1)	0.66	1.35 (0.89 to 2.03)	0.15	1.04 (0.88 to 1.24)	0.64	0.96 (0.74 to 1.24)	0.76	1.04 (0.93 to 1.18)	0.48
BODE		0.43 (0.3 to 0.56)	5.10E-11	0.38 (0.32 to 0.44)	4.10E-35	0.61 (0.39 to 0.83)	5.40E-08	0.19 (0.11 to 0.27)	3.70E-06	0.13 (-0.086 to 0.35)	0.24	0.053 (-0.051 to 0.16)	0.32
Dead	BODE	1.15 (0.96 to 1.36)	0.12	1.14 (1.05 to 1.24)	0.0016	1.06 (0.71 to 1.59)	0.76	1.22 (1.05 to 1.41)	0.0089	1.17 (0.94 to 1.44)	0.16	1.01 (0.92 to 1.12)	0.78
% LAA < -950 HU	CT scanner	0.37 (0.29 to 0.45)	1.10E-19	0.19 (0.15 to 0.23)	6.00E-24	0.32 (0.17 to 0.47)	4.30E-05	0.044 (-0.011 to 0.099)	0.11	0.12 (0.01 to 0.23)	0.032	0.11 (0.059 to 0.16)	3.80E-05
Perc15	CT scanner	−6.1 (-7.6 to -4.6)	1.20E-15	−3.4 (-4.1 to -2.7)	1.10E-21	−5.9 (-9.2 to -2.6)	0.00039	−1.4 (-2.6 to -0.24)	0.015	−2.4 (-5.3 to 0.54)	0.097	−2.5 (-3.8 to -1.2)	0.00028
Pi10	CT scanner	0.0092 (0.0016 to 0.017)	0.017	0.013 (0.0095 to 0.017)	2.30E-13	−0.0073 (-0.022 to 0.0072)	0.32	0.0069 (0.0018 to 0.012)	0.0091	−0.00037 (−0.018 to 0.017)	0.97	0.0034 (−0.0046 to 0.011)	0.4
WA%	CT scanner	0.35 (0.17 to 0.53)	0.00015	0.75 (0.67 to 0.83)	5.00E-66	0.29 (−0.082 to 0.66)	0.11	0.51 (0.38 to 0.64)	3.10E-14	0.15 (−0.28 to 0.58)	0.5	0.82 (0.62 to 1)	6.40E-16
Paraseptal emphysema	CT scanner	1.57 (1.17 to 2.11)	0.0029	1.5 (1.29 to 1.74)	6.20E-08	1.68 (1.04 to 2.7)	0.034	1.05 (0.88 to 1.27)	0.57	NA	NA	NA	NA
Bronchial airway disease	CT scanner	1.19 (0.83 to 1.71)	0.34	1.64 (1.38 to 1.95)	2.30E-08	0.77 (0.39 to 1.49)	0.44	1.25 (1 to 1.57)	0.051	NA	NA	NA	NA
Small airway disease	CT scanner	1.04 (0.68 to 1.57)	0.87	1.79 (1.47 to 2.17)	6.00E-09	1.71 (0.71 to 4.14)	0.23	1.04 (0.72 to 1.52)	0.82	NA	NA	NA	NA
Mild CLE	CT scanner	1.57 (1.22 to 2.03)	0.00053	1.41 (1.25 to 1.6)	4.20E-08	0.83 (0.48 to 1.43)	0.51	1.15 (0.95 to 1.39)	0.15	NA	NA	NA	NA
Upper lobe CLE	CT scanner	2.35 (1.46 to 3.77)	0.00042	1.83 (1.42 to 2.35)	2.50E-06	2.86 (0.94 to 8.73)	0.064	0.91 (0.52 to 1.58)	0.73	NA	NA	NA	NA
Lower lobe CLE	CT scanner	7.76 (2.19 to 27.41)	0.0015	2.41 (1.3 to 4.47)	0.005	*	*	*	*	NA	NA	NA	NA
Diffuse CLE	CT scanner	2.42 (1.64 to 3.57)	9.10E-06	2 (1.63 to 2.45)	3.70E-11	0.86 (0.27 to 2.72)	0.79	0.88 (0.56 to 1.39)	0.59	NA	NA	NA	NA
Visual without quantitative emphysema	CT scanner	2.12 (1.39 to 3.24)	0.00051	1.65 (1.34 to 2.03)	2.50E-06	1.31 (0.64 to 2.68)	0.46	1.22 (0.89 to 1.67)	0.21	NA	NA	NA	NA
Quantitative without visual emphysema	CT scanner	1.66 (0.87 to 3.17)	0.13	1.54 (1.11 to 2.13)	0.01	*	*	*	*	NA	NA	NA	NA

All models had form outcome ~family history+PRS+age+sex + pack-years+principal components of genetic ancestry+C, where C equals any additional covariates listed in the table for a specific outcome.^21^ % LAA ≤950 HU=per cent low attenuation area of the lung less than −950 HU. Perc15=15th percentile of the lung density histogram on inspiratory scans. Pi10=square root of wall area of a hypothetical airway with an internal perimeter of 10 mm. The PRS was treated as a continuous variable, and ORs are associated with 1 SD increase in the risk score.

*Indicates that model did not converge as certain phenotypes had few numbers of participants.

BODE, body mass index, obstruction, dyspnoea, exercise capacity index; CLE, centrilobular emphysema; FEV1, forced expiratory volume in 1 s; HU, Hounsfield units; LAA, low attenuation area; PRS, Polygenic Risk Score; SGRQ, St. George Respiratory Questionnaire; WA%, mean wall area per cent.

**Figure 3:**
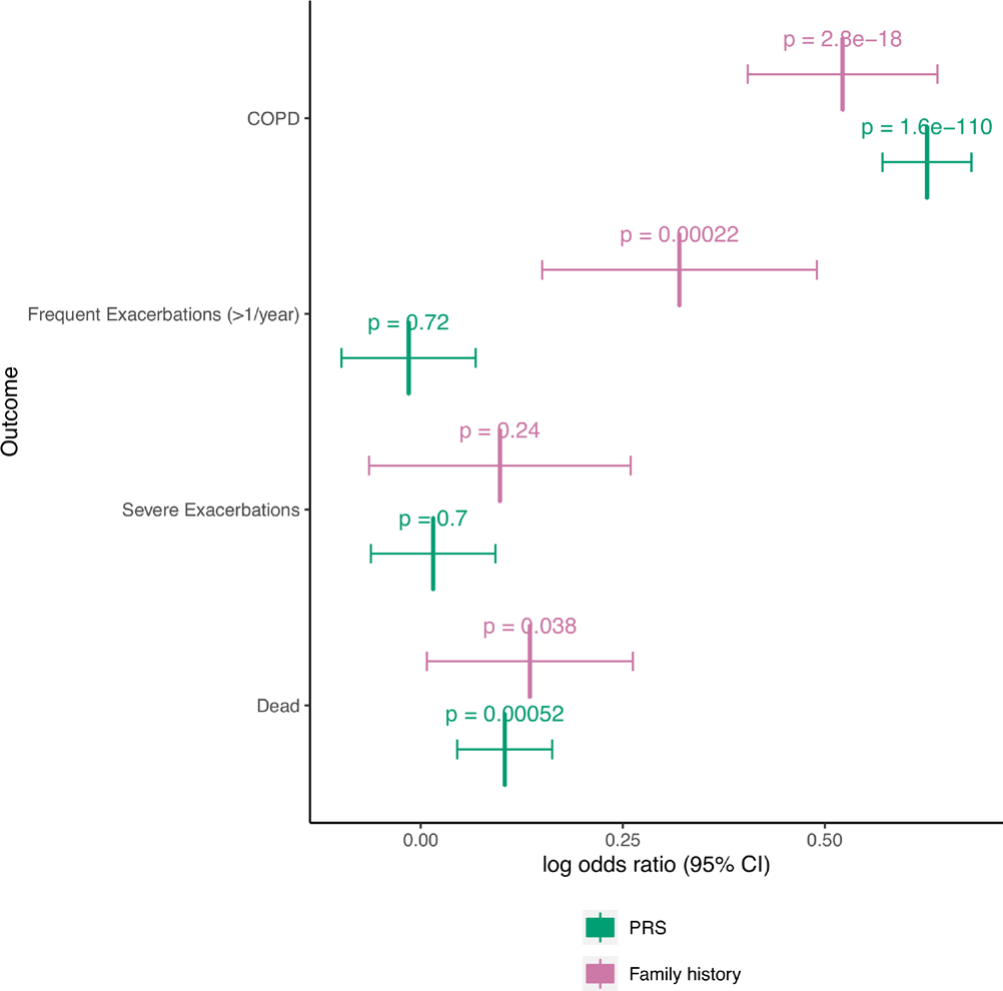
Meta-analyses of binary outcomes with PRS treated as a continuous variable. COPDGene and ECLIPSE studies were meta-analysed, and fixed effects odds ratios with 95% CIs are shown for family history and PRS for each outcome. ORs for the PRS indicate the odds ratio for the listed outcome for every standard deviation increase in the PRS. The Bonferroni-corrected level of significance is 0.05/11 = 0.0045 (includes seven continuous outcomes shown in supplement). Abbreviations are listed in the caption of table 3.

We found similar results in ECLIPSE (online supplemental figures S4 and S5). Model 2 demonstrated significantly improved performance over model 1, but model 3 performed similarly to model 2 for COPD prediction. For COPD-related traits, results were similar to COPDGene for all outcomes except for WA % and Pi10 in ECLIPSE. Results are also presented as a meta-analysis (figure 3, online supplemental figures S6‒S8).

### Analysis of interaction between family history and the PRS

As gene–gene and gene–environment interactions could lead to excess disease risk, we sought to understand whether having both a family history and higher PRS was associated with a greater OR for COPD than would be explained by either risk factor alone. Using the COPDGene study, we investigated whether there is an interaction between family history and the PRS. The OR for COPD for any given PRS value, separated by family history of COPD, is shown in online supplemental figure S9. In a logistic regression model of COPD, the family history * PRS interaction term was not significant on the multiplicative scale (COPDGene NHW: β=−0.09 (SE: 0.08), p=0.3; COPDGene AA: β=0.031 (SE: 0.12), p=0.8). Evaluation of the joint effects (online supplemental table S11) and stratified analyses (online supplemental table S12) of family history and the dichotomised PRS also suggest there is not a significant multiplicative interaction. The RERI was 0.48 (95% CI −0.04 to 1.00, p=0.04) in COPDGene NHW and 0.42 (95% CI −0.2 to 1.05, p=0.091) in COPDGene AA participants, suggesting some significant interaction on the additive scale in NHW participants.

### The effect of family history is partially mediated through PRS

We employed a natural effects model to investigate whether, and to what extent, the effects of family history are mediated through the PRS. The directed acyclic graph on which this analysis was based is shown in online supplemental figure S10. There was a significant NDE of family history on COPD in COPDGene NHW (β=0.48, p=2.0e-13) and AA (β=0.52, p=5.4e-6) participants. The NIE of family history on COPD through the PRS was significant in NHWs (β=0.09, p=8.7e-6), but not in AAs (β=0.017, p=0.5) in COPDGene. The proportion of the effect of family history mediated through the PRS was 16.5% (95% CI 9.4% to 24.3%) in COPDGene NHW and 3.1% (95% CI −6.5% to 11.9%) in COPDGene AA participants.

## Discussion

In this study of 9500 participants from two COPD case–control cohorts, we compared the relative effects of family history and a PRS on COPD and related clinical and chest CT quantitative imaging phenotypes. We observed that family history and PRS provide complementary information in association with COPD and COPD-related phenotypes. Moreover, we demonstrated that a PRS based on genotyping of SNPs is more predictive of COPD than family history in NHW and the predictive capacity of the PRS is comparable to family history in AA; this is an important milestone for genetic predictors. In NHWs, we also showed that approximately 17% of the effect of family history on COPD is mediated through the PRS. These data highlight the relative strengths and limitations of both family history and the PRS as predictors, and provide evidence that both genome-wide SNP genotyping and family history can contribute to an understanding of an individual’s risk for COPD. While comparative analyses of family history and PRSs have been performed in cardiovascular^16 17^ and psychiatric diseases,^18^ to our knowledge, this is the first analysis of the relative contributions of family history and a PRS to COPD risk.

Our findings are consistent with prior reports of the effect of positive family history on risk to COPD.^8 38 39^ In a prior study in COPDGene (limited to the first 2500 participants), a family history of COPD was associated with a 1.73 OR for the disease.^8^ Our study included more individuals and observed a similar effect size (OR (AA)=1.71 and OR (NHW)=1.77) with a similar population attributable risk in NHWs (15% vs 17%). The prior study did not analyse AA individuals separately, but we observe that the population attributable risk in COPDGene AAs is 8.7%.

Our findings have several implications. First, our findings suggest that family history and the PRS provide complementary information about an individual’s risk for COPD. We observed a statistically significant biserial correlation between family history and PRS in NHWs, though the strength of the correlation is small (correlation coefficient=0.093), suggesting that family history and PRS are primarily reflecting different aspects of COPD susceptibility; these results also imply that, contrary to traditional thought, family history does not appear to be a strong substitute for measured genetic risk in COPD. When comparing a model with both family history and the PRS to models with either predictor alone, we observed that the effect sizes and p values were largely unchanged, suggesting that the majority of the effects for family history and PRS do not lie on the same causal pathway; rather, complementary information regarding disease risk is gleaned from each measure. As family history must be a composite measure for genetics and shared environment, the residual effect of family history on COPD after adjusting for the PRS is presumably driven by shared environmental effects, or genetic factors not included or inaccurately modelled by the PRS. On the other hand, family history can only capture contributions from those whose parents developed and were aware of their diagnosis of disease, while the PRS is able to capture disease risk in the absence of any knowledge of parental disease. Our findings are also consistent with the current thinking that most complex disease cases are sporadic, occurring in individuals without a family history of the disease,^9^ and emphasise the potential predictive value added by obtaining a PRS for individual patients.

Second, we observe that for COPD itself, and for certain related quantitative phenotypes, the PRS has more explanatory power than family history and vice versa. The relative contribution of PRS versus family history depends on factors such as the relative contribution of the environment, the prevalence of the disease^40^ and the accuracy of reported family history. The PRS had comparable or larger effect sizes on risk to COPD affection status compared with family history. We estimated the attributable percent in the exposed for family history to be ~40% in all cohorts, implying that if no one had a family history of COPD, 40% fewer cases would have occurred. By contrast, the PRS had a population attributable risk of 18%–30% and attributable per cent in the exposed of ~70% in NHW and 45% in AA participants; therefore, this European-derived PRS can account for more of the risk of COPD than family history in NHWs, and is similar to family history in AAs. For COPD exacerbations, we confirm^8^ an association between family history and frequent exacerbations (ie, ≥2 exacerbations in the previous 12 months), after adjusting for baseline FEV_1_% predicted in the COPDGene study, with a trend towards association between family history and frequent exacerbations in ECLIPSE. Whether this association represents shared environmental or genetic effects is not clear, particularly since PRS itself was not associated with frequent exacerbations. Only a few studies have examined genetic risk to COPD exacerbations, and no studies to our knowledge have studied the heritability of exacerbations. Future investigations into the genetics of COPD-related exacerbations and the role of shared environment may clarify these issues.

Third, we demonstrate that, in NHWs, family history is associated with risk to COPD through a direct effect as well as an indirect effect mediated via PRS. This finding is consistent with reports that adoptees with at least one parent with COPD are more likely to have COPD than adoptees with no parents with COPD.^38^ The estimates in NHWs suggests that a significant proportion of the effect of family history on COPD can be explained by genetics, as measured by the PRS. We note that the model assumes no unmeasured confounding, but the proposed model does account for the major confounders considered in studies of COPD genetics. The difference in mediation analysis results between NHWs and AAs is likely explained by the fact that the PRS was derived in European ancestry individuals, and the linkage structure and allelic frequencies in African ancestry populations is not well reflected by the PRS. Together, these issues highlight the need for PRSs derived from cohorts that include African ancestry individuals, and the importance of performing genetic studies in multi-ancestry populations.

In NHW, we report ~17% of the effect of family history on COPD susceptibility is mediated through genetic variation, summarised by the PRS. Our finding is similar to the partial mediation of the effects of family history and a genetic risk score in predicting risk to schizophrenia.^18^ However, it is important to note that the indirect effect can be underestimated if there is measurement error associated with the mediator (ie, the PRS), and overestimated if there is measurement error associated with the exposure (ie, family history). Given the challenges of accurate diagnosis of COPD and the possibility of recall bias, we suspect family history may be particularly error-prone. Thus, the actual proportion of COPD risk due to family history mediated through the PRS is likely to be less than what we estimated. Regardless, our analyses suggest that a substantial component of the effect of family history on COPD could be due to shared environmental factors.

We also observed an interaction between family history and the PRS on the additive scale (but not multiplicative scale) in NHWs. Thus, on an additive scale, those with both a family history and a high PRS are at higher risk than would be predicted by each risk factor independently. Though this interaction was statistically significant, the effect was modest and thus the interaction between family history and the PRS may not be clinically relevant. Furthermore, there was not a statistically significant interaction in AAs on the additive scale.

Our study is the first to our knowledge that places family history of COPD in the context of a PRS. Compared with prior studies of family history in COPD, our sample size is larger and tests additional related phenotypes, including CT imaging. However, our study has several limitations. The study was performed in enriched cohorts of heavy smokers at high risk for COPD, and it is not clear what the benefit of collecting both family history and genotyping would be in the general population. Detailed questions about COPD history (which include COPD, chronic bronchitis and emphysema) are not routinely obtained in most population-based studies. Family history is particularly susceptible to recall bias, and is often unknown or incomplete. Despite this, we observed significant effects of family history on multiple outcomes. For COPD affection status, the AUC for a model with PRS and family history is 0.803 compared with an AUC of 0.798 for a model with PRS and no family history in NHWs; while this difference was statistically significant, the practical difference between these models is not compelling. However, in some cases, family history improves prediction of certain COPD-related phenotypes (eg, frequent exacerbations), which suggests that including both measures in a single model helps to account for COPD heterogeneity, as well as whether a person develops a diagnosis of moderate-to-severe COPD. Some of our findings failed to replicate in ECLIPSE, potentially due to reduced power. All participants were smokers, making it difficult to tease out the contribution of shared environment on the effects of family history; however, future studies including never smokers could help address these issues. The PRS was based on common variants, and therefore, the contribution of rare variants to COPD risk and the overlap with family history was not assessed in the current study. Finally, there is arguably no proven strategy for prevention of COPD aside from smoking cessation, even in high-risk individuals (based on genetic markers). Potentially, certain high-risk occupations could be avoided. Further investigations are required to address the clinical impact of knowing one’s family history and measured genetic risk for COPD.

In conclusion, we demonstrate how family history and the PRS provide complementary information for predicting COPD and related phenotypes, though the PRS appears to be an overall stronger predictor than family history alone. Future studies should examine the impact of using information from both measures on clinical practice.
