# Supplementary material for: Relative contributions of family history and a polygenic risk score on COPD and related outcomes: COPDGene and ECLIPSE studies
Source: BMJ Open Respir Res. 2020 Nov 25;7(1):e000755. doi: 10.1136/bmjresp-2020-000755 (PMC7689586; doi:10.1136/bmjresp-2020-000755)
Supplement: Supplementary data [file bmjresp-2020-000755supp001.pdf]

Supplementary Note

Table of Contents

Individual Study Information…………………………………………………………………2
Funding and Acknowledgements……………………………………………………………..3
Supplementary Tables
Table S1…………………………………………………………………………...……8
Table S2……………………………………………………………………………..9
Table S3……………………………………………………………………………...10
Table S4……………………………………………………………………………...11
Table S5……………………………………………………………………………...12
Table S6……………………………………………………………………………...13
Table S7……………………………………………………………………………...14
Table S8……………………………………………………………………………...16
Table S9……………………………………………………………………………...17
Table S10…...………………………………………………………………………...18
Table S11……..……………………………………………………………………...19
Table S12……..……………………………………………………………………...21
Supplementary Figures
Figure S1……………………………………………………………………………..22
Figure S2……………………………………………………………………………..24
Figure S3……………………………………………………………………………..26
Figure S4……………………………………………………………………………..28
Figure S5……………………………………………………………………………..30
Figure S6……………………………………………………………………………..32
Figure S7……………………………………………………………………………..34
Figure S8……………………………………………………………………………..35
Figure S9……………………………………………………………………………..37
Figure S10..…………………………………………………………………………..39

References……………………………………………………………………………………40

Moll M, *et al. BMJ Open Resp Res* 2020; 7:e000755. doi: 10.1136/bmjresp-2020-000755

Individual Study Information

This section describes each study and provides details about measurements and genotyping. All participants gave informed consent and study protocols were approved by local Research Ethics Committees and Institutional Review Boards.

COPDGene

COPDGene is a multicenter observational study which primarily consists of smokers with and without COPD[1]. Subjects in COPDGene have at least 10 pack-years of smoking (except for a smaller group of nonsmoking controls, who were excluded from the analyses in this manuscript). Illumina (San Diego, CA) performed genotyping on the HumanOmniExpress array. Genotyping at the Z and S alleles was performed in all subjects. Subjects with severe alpha-1 antitrypsin deficiency were excluded. Imputation was performed the Michigan Imputation Server to the Haplotype Resource Consortium[2] and 1000 Genomes Phase I v3 Cosmopolitan reference panels, for whites and African Americans, respectively. Variants with an r2 value of ≤ 0.3 were removed. Family history was obtained by questionnaire, asking as separate questions, whether there was a paternal family history of COPD, chronic bronchitis, or emphysema, and again for maternal family history (6 questions total).

ECLIPSE

The Evaluation of COPD Longitudinally to Identify Predictive Surrogate End-points (ECLIPSE) study was a case-control study of smokers with ≥ 10 pack years of smoking history, aged 40-75 years, and without other respiratory diseases.[3] Genotyping was performed using the Illumina HumanHap 550 V3 (Illumina, San Diego, CA). Subjects and markers with a call rate of < 95% were excluded. Imputation was performed using the Michigan Imputation Server and Haplotype Resource Consortium[2] reference panel. Family history was obtained by the American Thoracic Society Division of Lung Disease questionnaire (ATS-DLD-78A), which asked about a paternal or maternal family history of chronic bronchitis or emphysema (4 questions total).

Moll M, *et al. BMJ Open Resp Res* 2020; 7:e000755. doi: 10.1136/bmjresp-2020-000755

Funding and Acknowledgements

The International COPD Genetics Consortium: *Executive Committee*: James D. Crapo, William MacNee, David Lynch, H. Marike Boezen, Edwin K. Silverman, Jørgen Vestbo. *Members*: Alvar Agusti, Wayne Anderson, Nawar Bakerly, Per Bakke, Robert Bals, Kathleen C. Barnes, R Graham Barr, Terri H. Beaty, Eugene R. Bleecker, Yohan Bossé, Russell Bowler, Christopher Brightling, Marleen de Bruijne, Peter J. Castaldi, Bartolome Celli, Michael H. Cho, Harvey O. Coxson, Ron Crystal, Pim de Jong, Asger Dirksen, Jennifer Dy, Marilyn Foreman, Judith Garcia-Aymerich, Pierre Gevenois, Soumitra Ghosh, Hester Gietema, Amund Gulsvik, Ian P. Hall, Nadia Hansel, Craig P. Hersh, Brian D. Hobbs, Eric Hoffman, Noor Kalsheker, Hans-Ulrich Kauczor, Woo Jin Kim, Deog Kyeom Kim, Tarja Laitinen, Diether Lambrechts, Sang-Do Lee, Augusto A. Litonjua, David A. Lomas, Stephanie J. London, Daan W. Loth, Sharon M. Lutz, Merry-Lynn McDonald, Deborah A. Meyers, John D. Newell, Borge G. Nordestgaard, George T. O'Connor, Ma'en Obeidat, Yeon-Mok Oh, Peter D. Paré, Massimo Pistolesi, Dirkje S. Postma, Milo Puhan, Elizabeth Regan, Stephen S. Rich, Joon Beom Seo, Andrea Short, David Sparrow, Berend Stoel, David P. Strachan, Nicola Sverzellati, Ruth Tal-Singer, Gerben ter Riet, Yohannes Tesfaigzi, Martin D. Tobin, Edwin J.R. Van Beek, Bram van Ginneken, Claus F. Vogelmeier, Louise V. Wain, Adam Wanner, George Washko, Els Wauters, Emiel FM Wouters, Robert P. Young, and Loems Zeigler-Heitbrock.
The ICGC extends special thanks to Nora Franceschini, Kari North, Steve Rich, Xin-Qun Wang, Andre Uitterlinden, Bruno Stricker, Arfan Ikram, Megan Hardin, Gus Litonjua, Nick Locantore, Josée Dupuis, Elizabeth Ampleford, Eugene Bleecker, Yeon- Mok Oh, Shuguang Leng, Bruce Psaty, Susan Heckbert, and Jerry Rotter.

COPDGene Funding and Acknowledgements

Grant Support and Disclaimer

The project described was supported by Award Number U01 HL089897 and Award Number U01 HL089856 from the National Heart, Lung, and Blood Institute. The content is solely the responsibility of the authors and does not necessarily represent the official views of the National Heart, Lung, and Blood Institute or the National Institutes of Health.

COPD Foundation Funding

The COPDGene® project is also supported by the COPD Foundation through contributions made to an Industry Advisory Board comprised of AstraZeneca, Boehringer Ingelheim, GlaxoSmithKline, Novartis, Pfizer, Siemens and Sunovion.

COPDGene® Investigators – Core Units

Administrative Center: James D. Crapo, MD (PI); Edwin K. Silverman, MD, PhD (PI); Barry J. Make, MD; Elizabeth A. Regan, MD, PhD

Genetic Analysis Center: Terri Beaty, PhD; Ferdouse Begum, PhD; Peter J. Castaldi, MD, MSc; Michael Cho, MD; Dawn L. DeMeo, MD, MPH; Adel R. Boueiz, MD; Marilyn G. Foreman, MD, MS; Eitan Halper-Stromberg; Lystra P. Hayden, MD, MMSc; Craig P. Hersh, MD, MPH; Jacqueline Hetmanski, MS, MPH; Brian D. Hobbs, MD; John E. Hokanson, MPH, PhD; Nan Laird, PhD; Christoph Lange, PhD; Sharon M. Lutz, PhD; Merry-Lynn McDonald, PhD; Margaret M. Parker, PhD; Dandi Qiao, PhD; Elizabeth A. Regan, MD, PhD; Edwin K. Silverman, MD, PhD; Emily S. Wan, MD; Sungho Won, Ph.D.; Phuwanat Sakornsakolpat, M.D.; Dmitry Prokopenko, Ph.D.

Imaging Center: Mustafa Al Qaisi, MD; Harvey O. Coxson, PhD; Teresa Gray; MeiLan K. Han, MD, MS; Eric A. Hoffman, PhD; Stephen Humphries, PhD; Francine L. Jacobson, MD, MPH; Philip F. Judy, PhD; Ella A. Kazerooni, MD; Alex Kluiber; David A. Lynch, MB; John D. Newell, Jr., MD; Elizabeth A. Regan, MD, PhD; James C. Ross, PhD; Raul San Jose Estepar, PhD; Joyce Schroeder, MD; Jered Sieren; Douglas Stinson; Berend C. Stoel, PhD; Juerg Tschirren, PhD; Edwin Van Beek, MD, PhD; Bram van Ginneken, PhD; Eva van Rikxoort, PhD; George Washko, MD; Carla G. Wilson, MS;

PFT QA Center, Salt Lake City, UT: Robert Jensen, PhD

Data Coordinating Center and Biostatistics, *National Jewish Health, Denver, CO*: Douglas Everett, PhD; Jim Crooks, PhD; Camille Moore, PhD; Matt Strand, PhD; Carla G. Wilson, MS

Epidemiology Core, *University of Colorado Anschutz Medical Campus, Aurora, CO*: John E. Hokanson, MPH, PhD; John Hughes, PhD; Gregory Kinney, MPH, PhD; Sharon M. Lutz, PhD; Katherine Pratte, MSPH; Kendra A. Young, PhD

Mortality Adjudication Core: Surya Bhatt, MD; Jessica Bon, MD; MeiLan K. Han, MD, MS; Barry Make, MD; Carlos Martinez, MD, MS; Susan Murray, ScD; Elizabeth Regan, MD; Xavier Soler, MD; Carla G. Wilson, MS

Biomarker Core: Russell P. Bowler, MD, PhD; Katerina Kechris, PhD; Farnoush Banaei-Kashani, Ph.D

COPDGene® Investigators – Clinical Centers

Ann Arbor VA: Jeffrey L. Curtis, MD; Carlos H. Martinez, MD, MPH; Perry G. Pernicano, MD

Baylor College of Medicine, Houston, TX: Nicola Hanania, MD, MS; Philip Alapat, MD; Mustafa Atik, MD; Venkata Bandi, MD; Aladin Boriek, PhD; Kalpatha Guntupalli, MD; Elizabeth Guy, MD; Arun Nachiappan, MD; Amit Parulekar, MD;

Brigham and Women's Hospital, Boston, MA: Dawn L. DeMeo, MD, MPH; Craig Hersh, MD, MPH; Francine L. Jacobson, MD, MPH; George Washko, MD

Columbia University, New York, NY: R. Graham Barr, MD, DrPH; John Austin, MD; Belinda D'Souza, MD; Gregory D.N. Pearson, MD; Anna Rozenshtein, MD, MPH, FACR; Byron Thomashow, MD

Duke University Medical Center, Durham, NC: Neil MacIntyre, Jr., MD; H. Page McAdams, MD; Lacey Washington, MD

HealthPartners Research Institute, Minneapolis, MN: Charlene McEvoy, MD, MPH; Joseph Tashjian, MD

Johns Hopkins University, Baltimore, MD: Robert Wise, MD; Robert Brown, MD; Nadia N. Hansel, MD, MPH; Karen Horton, MD; Allison Lambert, MD, MHS; Nirupama Putcha, MD, MHS

Los Angeles Biomedical Research Institute at Harbor UCLA Medical Center, Torrance, CA: Richard Casaburi, PhD, MD; Alessandra Adami, PhD; Matthew Budoff, MD; Hans Fischer, MD; Janos Porszasz, MD, PhD; Harry Rossiter, PhD; William Stringer, MD

Michael E. DeBakey VAMC, Houston, *TX*: Amir Sharafkhaneh, MD, PhD; Charlie Lan, DO

Minneapolis VA: Christine Wendt, MD; Brian Bell, MD

Morehouse School of Medicine, Atlanta, GA: Marilyn G. Foreman, MD, MS; Eugene Berkowitz, MD, PhD; Gloria Westney, MD, MS

National Jewish Health, Denver, CO: Russell Bowler, MD, PhD; David A. Lynch, MB

Reliant Medical Group, Worcester, MA: Richard Rosiello, MD; David Pace, MD

Temple University, Philadelphia, PA: Gerard Criner, MD; David Ciccolella, MD; Francis Cordova, MD; Chandra Dass, MD; Gilbert D'Alonzo, DO; Parag Desai, MD; Michael Jacobs, PharmD; Steven Kelsen, MD, PhD; Victor Kim, MD; A. James Mamary, MD; Nathaniel Marchetti, DO; Aditi Satti, MD; Kartik Shenoy, MD; Robert M. Steiner, MD; Alex Swift, MD; Irene Swift, MD; Maria Elena Vega-Sanchez, MD

University of Alabama, Birmingham, AL: Mark Dransfield, MD; William Bailey, MD; Surya Bhatt, MD; Anand Iyer, MD; Hrudaya Nath, MD; J. Michael Wells, MD

University of California, San Diego, CA: Joe Ramsdell, MD; Paul Friedman, MD; Xavier Soler, MD, PhD; Andrew Yen, MD

University of Iowa, Iowa City, IA: Alejandro P. Comellas, MD; Karin F. Hoth, PhD; John Newell, Jr., MD; Brad Thompson, MD

University of Michigan, Ann Arbor, MI: MeiLan K. Han, MD, MS; Ella Kazerooni, MD; Carlos H. Martinez, MD, MPH

University of Minnesota, Minneapolis, MN: Joanne Billings, MD; Abbie Begnaud, MD; Tadashi Allen, MD

University of Pittsburgh, Pittsburgh, PA: Frank Sciurba, MD; Jessica Bon, MD; Divay Chandra, MD, MSc; Carl Fuhrman, MD; Joel Weissfeld, MD, MPH

University of Texas Health Science Center at San Antonio, San Antonio, TX: Antonio Anzueto, MD; Sandra Adams, MD; Diego Maselli-Caceres, MD; Mario E. Ruiz, MD

The **ECLIPSE** study (NCT00292552; GSK code SCO104960) was funded by GlaxoSmithKline.

ECLIPSE Investigators — *Bulgaria:* Y. Ivanov, Pleven; K. Kostov, Sofia. *Canada:* J. Bourbeau, Montreal; M. Fitzgerald, Vancouver, BC; P. Hernandez, Halifax, NS; K. Killian, Hamilton, ON; R. Levy, Vancouver, BC; F. Maltais, Montreal; D. O'Donnell, Kingston, ON. *Czech Republic:* J. Krepelka, Prague. *Denmark:* J. Vestbo, Hvidovre. *The Netherlands:*E. Wouters, Horn-

Maastricht. *New Zealand:* D. Quinn, Wellington. *Norway:* P. Bakke, Bergen. *Slovenia:* M. Kosnik, Golnik. *Spain:* A. Agusti, J. Sauleda, P. de Mallorca. *Ukraine:*Y. Feschenko, V. Gavrisyuk, L. Yashina, Kiev; N. Monogarova, Donetsk. *United Kingdom:* P. Calverley, Liverpool; D. Lomas, Cambridge; W. MacNee, Edinburgh; D. Singh, Manchester; J. Wedzicha, London. *United States:* A. Anzueto, San Antonio, TX; S. Braman, Providence, RI; R. Casaburi, Torrance CA; B. Celli, Boston; G. Giessel, Richmond, VA; M. Gotfried, Phoenix, AZ; G. Greenwald, Rancho Mirage, CA; N. Hanania, Houston; D. Mahler, Lebanon, NH; B. Make, Denver; S. Rennard, Omaha, NE; C. Rochester, New Haven, CT; P. Scanlon, Rochester, MN; D. Schuller, Omaha, NE; F. Sciurba, Pittsburgh; A. Sharafkhaneh, Houston; T. Siler, St. Charles, MO; E. Silverman, Boston; A. Wanner, Miami; R. Wise, Baltimore; R. ZuWallack, Hartford, CT.

ECLIPSE Steering Committee: H. Coxson (Canada), C. Crim (GlaxoSmithKline, USA), L. Edwards (GlaxoSmithKline, USA), D. Lomas (UK), W. MacNee (UK), E. Silverman (USA), R. Tal Singer (Co-chair, GlaxoSmithKline, USA), J. Vestbo (Co-chair, Denmark), J. Yates (GlaxoSmithKline, USA).

ECLIPSE Scientific Committee: A. Agusti (Spain), P. Calverley (UK), B. Celli (USA), C. Crim (GlaxoSmithKline, USA), B. Miller (GlaxoSmithKline, USA), W. MacNee (Chair, UK), S. Rennard (USA), R. Tal-Singer (GlaxoSmithKline, USA), E. Wouters (The Netherlands), J. Yates (GlaxoSmithKline, USA).

Supplementary Tables

Table S1: Unadjusted odds ratios for the association of family history and PRS with moderate-to-severe COPD in each cohort.

	**COPDGene NHW**		**COPDGene AA**		**ECLIPSE**	
Variable	*OR (95% CI)*	*p*	*OR (95% CI)*	*p*	*OR (95% CI)*	*p*
Family history	1.58 (1.4 - 1.78)	1.10E-13	1.68 (1.35 - 2.09)	2.80E-06	1.36 (0.95 - 1.93)	0.092
PRS	1.94 (1.82 - 2.07)	3.70E-90	1.41 (1.29 - 1.54)	3.60E-15	2 (1.66 - 2.4)	2.10E-13

Table S2: Associations of family history and PRS in three logistic regression models of moderate-to-severe COPD: Model 1 (COPD ~ Family history + age + pack years + sex); Model 2 (COPD ~ PRS + age + pack years + sex); Model 3 (COPD ~ family history + PRS + age + pack years + sex). Bonferroni-adjusted level of significance is 0.05/3 models = 0.017. The PRS was dichotomized (top vs bottom two tertiles).

**COPDGene NHW**						
	**Model 1**		**Model 2**		**Model 3**	
Variable	*OR (95% CI)*	*p-value*	*OR (95% CI)*	*p-value*	*OR (95% CI)*	*p-value*
Family history	1.77 (1.55 - 2.03)	4.30E-17	NA	NA	1.67 (1.45 - 1.92)	5.10E-13
PRS	NA	NA	3.45 (3 - 3.98)	7.30E-66	3.39 (2.94 - 3.91)	3.10E-63
**COPDGene AA**						
	**Model 1**		**Model 2**		**Model 3**	
	OR (95% CI)	*p-value*	*OR (95% CI)*	*p-value*	*OR (95% CI)*	*p-value*
Family history	1.71 (1.35 - 2.17)	9.50E-06	NA	NA	1.74 (1.37 - 2.21)	7.00E-06
PRS	NA	NA	1.8 (1.5 - 2.17)	6.40E-10	1.81 (1.5 - 2.18)	6.70E-10
**ECLIPSE**						
	**Model 1**		**Model 2**		**Model 3**	
	OR (95% CI)	*p-value*	*OR (95% CI)*	*p-value*	*OR (95% CI)*	*p-value*
Family history	1.33 (0.91 - 1.94)	0.14	NA	NA	1.71 (1.15 - 2.54)	0.0082
PRS	NA	NA	3.06 (1.89 - 4.94)	4.90E-06	3.05 (1.89 - 4.94)	5.50E-06

Moll M, *et al. BMJ Open Resp Res* 2020; 7:e000755. doi: 10.1136/bmjresp-2020-000755

Table S3: Participants who reported not knowing their family history for COPD were excluded from analyses, and revised demographics are shown below.

	**COPDGene NHW**		**COPDGene AA**		**ECLIPSE**	
	Controls	Cases	Controls	Cases	Controls	Cases
n	1998	2080	1175	487	147	1713
Age in years (mean (SD))	59.76 (8.76)	64.63 (8.13)	53.13 (6.12)	59.72 (8.21)	57.32 (9.55)	63.64 (7.10)
Sex (No. female, (%))	1049 (52.5)	940 (45.2)	508 (43.2)	233 (47.8)	63 (42.9)	563 (32.9)
BMI (kg/m^2) (mean (SD))	28.77 (5.70)	27.90 (6.05)	29.24 (6.26)	27.92 (6.84)	27.34 (4.17)	26.53 (5.54)
Current Smoking (No. (%))	768 (38.4)	710 (34.1)	1018 (86.6)	276 (56.7)	46 (37.7)	475 (35.4)
Pack-years cigarette smoking (mean (SD))	38.01 (20.50)	55.83 (27.16)	36.56 (19.89)	42.82 (23.00)	31.01 (25.94)	50.50 (27.47)
SGRQ Total Score (mean (sd)) (mean (SD))	16.25 (16.55)	40.52 (21.09)	23.08 (20.00)	45.02 (23.27)	9.45 (13.11)	50.91 (19.89)
6-minute walk distance (ft) (mean (SD))	1573.42 (326.40)	1216.65 (384.36)	1362.30 (355.35)	1022.49 (395.50)	NA	1084.17 (355.24)
BODE (mean (SD))	0.34 (0.77)	2.92 (2.05)	0.74 (1.08)	3.16 (2.08)	NA	3.26 (2.13)
Frequent Exacerbations (No (%) > 1 per year)	37 (2.5)	181 (15.0)	30 (4.7)	34 (13.9)	0 (0.0)	775 (45.7)
Severe Exacerbations (No. (%))	50 (3.4)	230 (19.1)	52 (8.1)	63 (25.7)	0 (0.0)	542 (31.6)
FEV1 % predicted (mean (SD))	95.21 (12.96)	48.96 (17.93)	96.32 (14.68)	50.89 (18.25)	108.87 (12.28)	47.11 (15.47)
FEV1/FVC ratio (mean (SD))	0.77 (0.06)	0.48 (0.13)	0.79 (0.06)	0.52 (0.12)	0.80 (0.05)	0.44 (0.11)
Combined FEV1 and FEV1/FVC PRS (mean (sd)) (mean (SD))	-0.26 (0.97)	0.25 (0.96)	-0.07 (0.99)	0.20 (1.03)	-0.60 (0.98)	0.05 (0.98)
Family history of COPD, chronic bronchitis, or emphysema	710 (35.5)	991 (47.6)	247 (21.0)	145 (29.8)	51 (34.7)	717 (41.9)

Moll M, *et al. BMJ Open Resp Res* 2020; 7:e000755. doi: 10.1136/bmjresp-2020-000755

Table S4: Participants who reported not knowing their family history for COPD were excluded from analyses, and shown are the associations of family history and PRS in three logistic regression models of moderate-to-severe COPD: Model 1 (COPD ~ Family history + age + pack years + sex); Model 2 (COPD ~ PRS + age + pack years + sex); Model 3 (COPD ~ family history + PRS + age + pack years + sex).

	**COPDGene NHW**					
	**Model 1**		**Model 2**		**Model 3**	
Variable	*OR (95% CI)*	*p-value*	*OR (95% CI)*	*p-value*	*OR (95% CI)*	*p-value*
Family history	1.93 (1.67 - 2.23)	4.20E-19	NA	NA	1.81 (1.55 - 2.11)	3.50E-14
PRS	NA	NA	2.09 (1.93 - 2.26)	9.50E-72	2.07 (1.91 - 2.24)	8.80E-69
	**COPDGene AA**					
	**Model 1**		**Model 2**		**Model 3**	
	OR (95% CI)	*p-value*	*OR (95% CI)*	*p-value*	*OR (95% CI)*	*p-value*
Family history	1.91 (1.49 - 2.46)	4.50E-07	NA	NA	1.97 (1.52 - 2.55)	2.70E-07
PRS	NA	NA	1.47 (1.31 - 1.65)	2.10E-11	1.48 (1.32 - 1.65)	2.10E-11
	**ECLIPSE**					
	**Model 1**		**Model 2**		**Model 3**	
	OR (95% CI)	*p-value*	*OR (95% CI)*	*p-value*	*OR (95% CI)*	*p-value*
Family history	1.33 (0.91 - 1.94)	0.14	NA	NA	1.69 (1.13 - 2.53)	0.011
PRS	NA	NA	2.02 (1.65 - 2.47)	6.70E-12	2.01 (1.64 - 2.45)	8.70E-12

Table S5: The definition of family history of COPD in COPDGene was harmonized with ECLIPSE, considering a maternal or paternal family history of chronic bronchitis or emphysema to indicate a positive family history of COPD. For COPDGene AA, 351 individuals had a positive family history of COPD, and for COPDGene NHW 1552 individuals had a positive family history of CODP. Below, the associations of family history and PRS are shown in three logistic regression models of moderate-to-severe COPD: Model 1 (COPD ~ Family history + age + pack years + sex); Model 2 (COPD ~ PRS + age + pack years + sex); Model 3 (COPD ~ family history + PRS + age + pack years + sex). ECLIPSE analyses were unchanged.

	**COPDGene NHW**						**COPDGene AA**					
	**Model 1**		**Model 2**		**Model 3**		**Model 1**		**Model 2**		**Model 3**	
Variable	*OR (95% CI)*	*p-value*	*OR (95% CI)*	*p-value*	*OR (95% CI)*	*p-value*	*OR (95% CI)*	*p-value*	*OR (95% CI)*	*p-value*	*OR (95% CI)*	*p-value*
Family history	1.82 (1.59 - 2.09)	1.30E-17	NA	NA	1.7 (1.46 - 1.96)	1.40E-12	1.64 (1.28 - 2.1)	9.30E-05	NA	NA	1.67 (1.3 - 2.16)	7.50E-05
PRS	NA	NA	2.13 (1.98 - 2.28)	9.00E-95	2.11 (1.96 - 2.27)	1.00E-91	NA	NA	1.5 (1.36 - 1.64)	2.50E-17	1.5 (1.36 - 1.65)	3.10E-17

Table S6: Measures of population risk for family history and PRS that has been dichotomized (top tertile versus bottom two tertiles). Odds ratios are adjusted for age, sex, pack-years of cigarette smoking, and principal components of genetic ancestry.

	**COPDGene NHW**		**COPDGene AA**		**ECLIPSE**	
Measure (point estimate [95% CI])	*Family history*	*PRS (top tertile vs bottom 2 tertiles)*	*Family history*	*PRS (top tertile vs bottom 2 tertiles)*	*Family history*	*PRS (top tertile vs bottom 2 tertiles)*
Attributable fraction in the exposed	0.4 (0.31-0.48)	0.7 (0.66-0.74)	0.42 (0.27-0.55)	0.45 (0.33-0.54)	0.41 (0.13-0.61)	0.67 (0.47-0.8)
Odds ratio	1.7 (1.4-1.8)	3.4 (2.5-3.3)	1.7 (1.3-2.1)	1.8 (1.3-1.9)	1.7 (0.91-2)	3.1 (1.8-4.7)
Population attributable fraction	0.15 (0.087-0.23)	0.29 (0.21-0.41)	0.087 (0.034-0.18)	0.18 (0.094-0.32)	0.17 (0.024-0.56)	0.23 (0.063-0.73)

Moll M, *et al. BMJ Open Resp Res* 2020; 7:e000755. doi: 10.1136/bmjresp-2020-000755

Table S7: Association of family history and PRS with outcomes. All models had form Outcome ~ family history + PRS + age + sex + pack years + C, where C equals any additional covariates listed in the table for a specific outcome. The PRS was dichotomized (top vs. bottom two tertiles). CLE = centrilobular emphysema. BODE = body-mass, obstructive, dyspnea, exercise capacity index [4]. SGRQ = St. George Respiratory Questionnaire. % LAA < -950 HU = percent low attenuation area of the lung less than -950 Hounsfield units. Perc15 = 15th percentile of the lung density histogram on inspiratory scans. Pi10 = square root of wall area of a hypothetical airway with an internal perimeter of 10 mm. WA % = mean wall area percent. ** indicates model did not converge as certain phenotypes had few numbers of participants.

Outcome	Covariates	**COPDGene NHW**				**COPDGene AA**				**ECLIPSE**			
		Family history (OR or beta (95% CI))	*p*	*PRS (OR or beta (95% CI))*	*p*	*Family history (OR or beta (95% CI))*	*p*	*PRS (OR or beta (95% CI))*	*p*	*Family history (OR or beta (95% CI))*	*p*	*PRS (OR or beta (95% CI))*	*p*
6-minute walk distance	height, weight	-27 (-49--5.4)	0.015	-64 (-86--42)	5.40E-09	-37 (-76-2.2)	0.073	-38 (-69--6.6)	0.013	-29 (-66-8.2)	0.14	-16 (-53-21)	0.41
SGRQ Total Score		0.28 (0.22-0.34)	2.70E-17	0.22 (0.16-0.28)	3.50E-12	0.39 (0.26-0.52)	4.70E-09	0.13 (0.03-0.23)	0.012	3.7 (1.5-5.9)	0.00067	3.1 (0.94-5.3)	0.0052
Frequent Exacerbations	FEV1 % predicted, current smoking	1.66 (1.26 - 2.18)	0.00026	0.77 (0.58 - 1.03)	0.075	1.37 (0.82 - 2.31)	0.23	1.1 (0.69 - 1.74)	0.69	1.19 (0.94 - 1.52)	0.15	1.04 (0.82 - 1.33)	0.73
Severe Exacerbations	FEV1 % predicted, current smoking	1.17 (0.92 - 1.49)	0.21	0.9 (0.7 - 1.15)	0.39	1.34 (0.89 - 2.02)	0.16	1 (0.7 - 1.43)	0.99	0.96 (0.75 - 1.24)	0.76	1.03 (0.8 - 1.32)	0.85
BODE		0.4 (0.29-0.51)	4.50E-12	0.61 (0.5-0.72)	3.00E-27	0.57 (0.39-0.75)	1.20E-09	0.26 (0.12-0.4)	0.00022	0.13 (-0.086-0.35)	0.23	0.2 (-0.016-0.42)	0.068
Dead	BODE	1.14 (0.96 - 1.36)	0.12	1.31 (1.1 - 1.54)	0.0018	1.06 (0.71 - 1.58)	0.78	1.3 (0.96 - 1.76)	0.095	1.17 (0.94 - 1.45)	0.16	1.06 (0.85 - 1.31)	0.61

% LAA < -950 HU	CT scanner	0.38 (0.3-0.46)	4.50E-20	0.34 (0.26-0.42)	1.40E-16	0.32 (0.17-0.47)	4.20E-05	0.049 (-0.065-0.16)	0.4	0.12 (0.01-0.23)	0.026	0.19 (0.08-0.3)	0.00082
Perc15	CT scanner	-6.2 (-7.7--4.7)	5.40E-16	-5.9 (-7.4--4.4)	1.20E-14	-5.9 (-9.2--2.6)	0.00037	-1.6 (-4.1-0.95)	0.21	-2.6 (-5.5-0.34)	0.084	-4.4 (-7.3--1.5)	0.0028
Pi10	CT scanner	0.0093 (0.0017-0.017)	0.016	0.026 (0.019-0.033)	9.10E-12	-0.0071 (-0.022-0.0074)	0.34	0.013 (0.002-0.024)	0.021	-7.6e-05 (-0.018-0.017)	0.99	-0.004 (-0.021-0.013)	0.65
WA %	CT scanner	0.37 (0.19-0.55)	6.50E-05	1.3 (1.1-1.5)	1.40E-42	0.31 (-0.062-0.68)	0.1	0.94 (0.67-1.2)	2.60E-11	0.18 (-0.25-0.61)	0.4	1.2 (0.77-1.6)	6.30E-08
Paraseptal emphysema	CT scanner	1.59 (1.18 - 2.14)	0.0021	2.04 (1.51 - 2.76)	3.00E-06	1.69 (1.05 - 2.73)	0.03	1.27 (0.85 - 1.9)	0.25	NA	NA	NA	NA
Bronchial airway disease	CT scanner	1.22 (0.85 - 1.75)	0.27	2.49 (1.76 - 3.52)	2.90E-07	0.77 (0.39 - 1.5)	0.44	1.69 (1.04 - 2.75)	0.034	NA	NA	NA	NA
Small airway disease	CT scanner	1.05 (0.7 - 1.6)	0.8	2.92 (1.99 - 4.28)	4.70E-08	1.7 (0.71 - 4.12)	0.24	1.01 (0.47 - 2.16)	0.99	NA	NA	NA	NA
Mild CLE	CT scanner	1.6 (1.24 - 2.06)	0.00032	1.79 (1.38 - 2.33)	1.40E-05	0.83 (0.48 - 1.43)	0.51	1.46 (0.97 - 2.19)	0.073	NA	NA	NA	NA
Upper lobe CLE	CT scanner	2.4 (1.5 - 3.84)	0.00028	2.75 (1.68 - 4.5)	5.30E-05	3.09 (1.01 - 9.52)	0.049	1.49 (0.51 - 4.39)	0.46	NA	NA	NA	NA
Lower lobe CLE	CT scanner	7.99 (2.26 - 28.29)	0.0013	7.05 (1.9 - 26.15)	0.0035	**	**	**	**	NA	NA	NA	NA
Diffuse CLE	CT scanner	2.38 (1.62 - 3.5)	1.10E-05	3.24 (2.19 - 4.8)	4.80E-09	0.91 (0.29 - 2.86)	0.87	1.02 (0.42 - 2.46)	0.97	NA	NA	NA	NA
Visual without quantitative emphysema	CT scanner	2.17 (1.42 - 3.31)	0.00034	2.78 (1.81 - 4.25)	2.60E-06	1.28 (0.63 - 2.63)	0.5	1.54 (0.82 - 2.89)	0.18	NA	NA	NA	NA
Quantitative without visual emphysema	CT scanner	1.74 (0.91 - 3.31)	0.094	1.71 (0.89 - 3.3)	0.11	**	**	**	**	NA	NA	NA	NA

Table S8: P-values comparing AUCs of models trained for binary outcomes. Model 1 (Outcome ~ Family history + covariates); Model 2 (Outcome ~ PRS + covariates); Model 3 (Outcome ~ family history + PRS + covariates). "*" indicates p-values less than Bonferroni-adjusted levels of significance.

cohort	*outcome*	*P (Model 2 vs Model 1)*	*P (Model 3 vs Model 2)*	*P (Model 3 vs Model 1)*
COPDGene AA	Moderate-to-severe COPD	0.00025*	0.12	1e-06*
COPDGene AA	Frequent Exacerbations (>1/year)	0.24	0.25	0.058
COPDGene AA	Severe Exacerbations	0.63	0.15	0.099
COPDGene AA	Vital Status	0.13	0.99	0.13
COPDGene NHW	Moderate-to-severe COPD	8.4e-21*	0.00035*	6.1e-29*
COPDGene NHW	Frequent Exacerbations (>1/year)	0.45	0.021	0.065
COPDGene NHW	Severe Exacerbations	0.51	0.089	0.077
COPDGene NHW	Vital Status	0.093	0.44	0.032
ECLIPSE	Moderate-to-severe COPD	4.1e-06*	0.97	3.3e-06*
ECLIPSE	Vital Status	0.082	0.48	0.03
ECLIPSE	Frequent Exacerbations (>1/year)	0.0042*	0.17	2.7e-05*
ECLIPSE	Severe Exacerbations	0.0028*	0.84	0.0013*

Table S9: Scaled Brier scores[5,6] comparing models trained for binary outcomes in each cohort. Model 1 (Outcome ~ Family history + covariates); Model 2 (Outcome ~ PRS + covariates); Model 3 (Outcome ~ family history + PRS + covariates). A higher scaled Brier score indicates a better model fit and greater variance explained.

Outcome	*Cohort*	*Model 1*	*Model 2*	*Model 3*
Moderate-to-severe COPD	COPDGene AA	0.157	0.181	0.188
Moderate-to-severe COPD	COPDGene NHW	0.185	0.264	0.272
Moderate-to-severe COPD	ECLIPSE	0.148	0.212	0.223
Frequent Exacerbations (>1/year)	COPDGene AA	0.0101	0.0247	0.0299
Frequent Exacerbations (>1/year)	COPDGene NHW	0.0179	0.0138	0.0224
Frequent Exacerbations (>1/year)	ECLIPSE	0.0264	0.0492	0.0537
Severe Exacerbations	COPDGene AA	0.0181	0.0244	0.0289
Severe Exacerbations	COPDGene NHW	0.017	0.0235	0.025
Severe Exacerbations	ECLIPSE	0.00882	0.0211	0.0215
Dead	COPDGene AA	0.061	0.07	0.0703
Dead	COPDGene NHW	0.0738	0.0767	0.0774
Dead	ECLIPSE	0.0507	0.0592	0.0601

Table S10: Measures of model fit for continuous outcomes in COPDGene and ECLIPSE, including adjusted R^2 and means squared error (MSE). Model 1 (Outcome ~ Family history + covariates); Model 2 (Outcome ~ PRS + covariates); Model 3 (Outcome ~ family history + PRS + covariates). A higher adjusted R2 indicates more variance explained, and lower mean squared error indicates a better model fit. The PRS was treated as a continuous variable. Abbreviations are the same as listed in caption for Table S3. AA = "African Americans". NHW = "non-Hispanic whites".

		**Adjusted R2**			**MSE**		
cohort	*outcome*	*Model 1*	*Model 2*	*Model 3*	*Model 1*	*Model 2*	*Model 3*
COPDGene AA	6MWD	0.12	0.13	0.13	140000	140000	130000
COPDGene NHW	6MWD	0.15	0.16	0.16	140000	140000	140000
ECLIPSE	6MWD	0.025	0.063	0.064	120000	120000	120000
COPDGene AA	BODE	0.14	0.14	0.15	4	4	3.9
COPDGene NHW	BODE	0.13	0.16	0.16	4.9	4.8	4.7
ECLIPSE	BODE	0.00065	0.0069	0.0071	4.5	4.5	4.5
COPDGene AA	% LAA < -950 HU	0.16	0.16	0.17	2.1	2.1	2.1
COPDGene NHW	% LAA < -950 HU	0.18	0.19	0.2	2.1	2.1	2
ECLIPSE	% LAA < -950 HU	0.034	0.047	0.049	1.1	1.1	1.1
COPDGene AA	Perc15	0.16	0.15	0.16	890	890	890
COPDGene NHW	Perc15	0.14	0.15	0.16	690	680	670
ECLIPSE	Perc15	0.061	0.076	0.081	780	760	760
COPDGene AA	Pi10	0.048	0.051	0.051	0.016	0.016	0.016
COPDGene NHW	Pi10	0.051	0.065	0.066	0.016	0.016	0.016
ECLIPSE	Pi10	0.25	0.28	0.28	0.03	0.028	0.028
COPDGene AA	SGRQ	0.08	0.069	0.082	1.3	1.4	1.3
COPDGene NHW	SGRQ	0.11	0.12	0.13	1.1	1.1	1.1
ECLIPSE	SGRQ	0.013	0.03	0.035	480	470	470
COPDGene AA	WA %	0.03	0.056	0.057	11	11	11
COPDGene NHW	WA %	0.048	0.11	0.11	10	9.3	9.3
ECLIPSE	WA %	0.0064	0.063	0.063	17	16	16

Moll M, *et al. BMJ Open Resp Res* 2020; 7:e000755. doi: 10.1136/bmjresp-2020-000755

Table S11: Joint analyses of family history and a low versus high PRS. A categorical variable was derived comparing each of the groups to the reference group of "no family history, low PRS". Adjusted models included age, sex, pack-years of cigarette smoking, and principal components of genetic ancestry.

Unadjusted

	PRS (lowest tertiles)		PRS (highest tertile)	
**COPDGene AA**	*OR (95% CI)*	*p*	*OR (95% CI)*	*p*
No family history	1 (ref)	0	1.58 (1.31 - 1.91)	1.70E-06
Family history	1.69 (1.29 - 2.22)	0.00017	2.65 (1.86 - 3.77)	6.20E-08

**COPDGene NHW**	*OR (95% CI)*	*p*	*OR (95% CI)*	*p*
No family history	1 (ref)	0	2.7 (2.31 - 3.16)	8.80E-36
Family history	1.47 (1.27 - 1.7)	2.10E-07	4.35 (3.52 - 5.37)	2.40E-42

**ECLIPSE**	*OR (95% CI)*	*p*	*OR (95% CI)*	*p*
No family history	1 (ref)	0	3.69 (1.99 - 6.86)	3.50E-05
Family history	1.49 (1.01 - 2.22)	0.046	2.96 (1.55 - 5.65)	0.00096

Adjusted

	PRS (lowest tertiles)		PRS (highest tertile)	
**COPDGene AA**	*OR (95% CI)*	*p*	*OR (95% CI)*	*p*
No family history	1 (ref)	0	1.83 (1.49 - 2.25)	9.60E-09
Family history	1.78 (1.32 - 2.41)	0.00017	3.04 (2.07 - 4.47)	1.50E-08

Moll M, *et al. BMJ Open Resp Res* 2020; 7:e000755. doi: 10.1136/bmjresp-2020-000755

**COPDGene NHW**	*OR (95% CI)*	*p*	*OR (95% CI)*	*p*
No family history	1 (ref)	0	3.36 (2.82 - 4)	1.10E-42
Family history	1.66 (1.41 - 1.96)	1.70E-09	5.64 (4.48 - 7.1)	6.30E-49

**ECLIPSE**	*OR (95% CI)*	*p*	*OR (95% CI)*	*p*
No family history	1 (ref)	0	3.97 (2.07 - 7.62)	3.40E-05
Family history	1.93 (1.24 - 3.02)	0.0036	4.05 (2.03 - 8.09)	7.30E-05

Table S12: Stratified analyses of family history and the dichotomized PRS.

cohort	*stratum*	*predictor*	*OR (95% CI) [unadjusted)*	*p [unadjusted]*	*OR (95% CI) [adjusted]*	*p [adjusted]*
COPDGene AA	PRS lowest tertiles	Family history	1.69 (1.29 - 2.22)	0.00017	1.79 (1.32 - 2.43)	0.00017
COPDGene AA	PRS highest tertile	Family history	1.68 (1.16 - 2.42)	0.0056	1.67 (1.12 - 2.49)	0.011
COPDGene AA	No family history	PRS top tertile	1.58 (1.31 - 1.91)	1.70E-06	1.82 (1.48 - 2.24)	1.10E-08
COPDGene AA	Family history present	PRS top tertile	1.57 (1.03 - 2.38)	0.034	1.73 (1.09 - 2.76)	0.021
COPDGene NHW	PRS lowest tertiles	Family history	1.47 (1.27 - 1.7)	2.10E-07	1.69 (1.43 - 1.99)	8.20E-10
COPDGene NHW	PRS highest tertile	Family history	1.61 (1.27 - 2.04)	8.30E-05	1.66 (1.28 - 2.15)	0.00012
COPDGene NHW	No family history	PRS top tertile	2.7 (2.31 - 3.16)	8.80E-36	3.35 (2.82 - 3.99)	3.40E-42
COPDGene NHW	Family history present	PRS top tertile	2.96 (2.35 - 3.72)	3.00E-20	3.41 (2.65 - 4.39)	2.70E-21
ECLIPSE	PRS lowest tertiles	Family history	1.49 (1.01 - 2.22)	0.046	1.89 (1.21 - 2.95)	0.0051
ECLIPSE	PRS highest tertile	Family history	0.8 (0.35 - 1.85)	0.6	0.96 (0.38 - 2.45)	0.94
ECLIPSE	No family history	PRS top tertile	3.69 (1.99 - 6.86)	3.50E-05	4.1 (2.11 - 7.97)	3.10E-05
ECLIPSE	Family history present	PRS top tertile	1.98 (1 - 3.93)	0.05	2.02 (0.98 - 4.13)	0.056

Moll M, *et al. BMJ Open Resp Res* 2020; 7:e000755. doi: 10.1136/bmjresp-2020-000755

Supplementary Figures

Figure S1: Distribution of PRS values in individuals with (NHW: n=1,701, AA: n=392) and without (NHW: n=3,473, AA: 2,074) a family history of COPD in the COPDGene study.

A) Boxplot - the box represents the interquartile range, with the horizontal line in the middle representing the medial. The lines represent 1.5 times the interquartile range

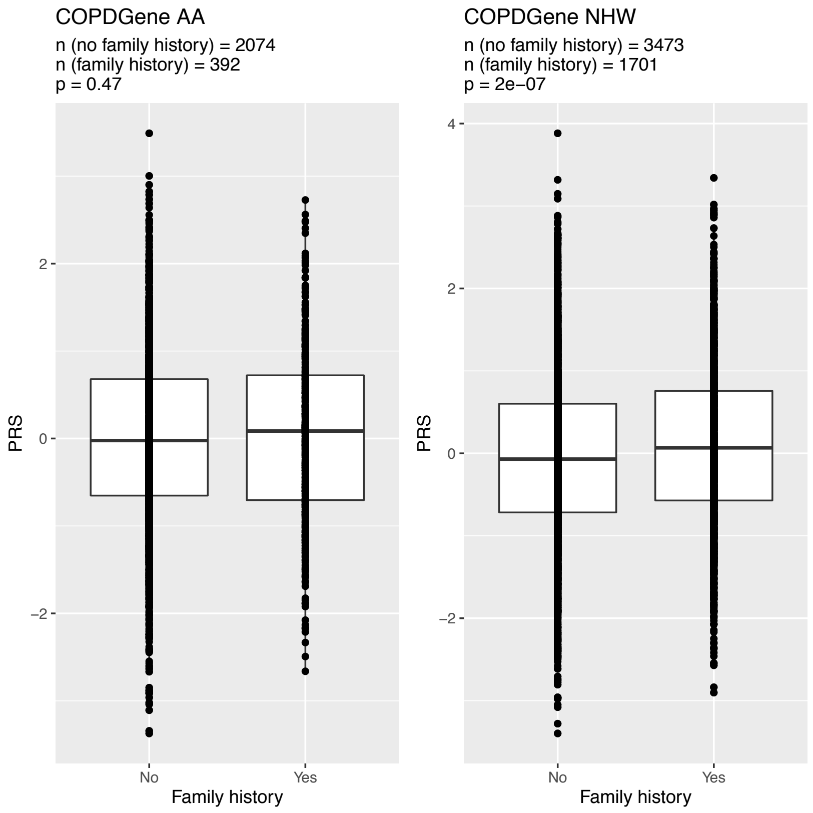

B) Density plots – COPDGene AA (*left*) and NHW (*right*)

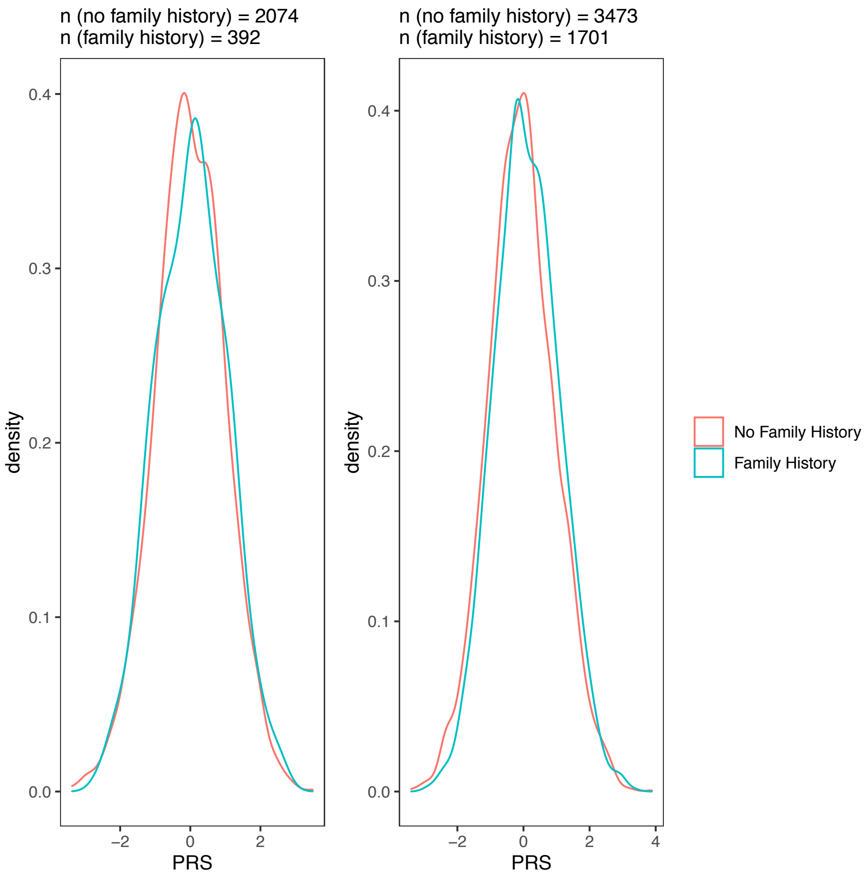

Moll M, *et al. BMJ Open Resp Res* 2020; 7:e000755. doi: 10.1136/bmjresp-2020-000755

Figure S2: A) COPDGene NHW: Predictive performance (adjusted R^2) of three linear regression models for outcomes shown on the *x-axis*. For each outcome, three models were trained: Model 1 (Outcome ~ family history + age + sex + pack-years), Model 2 (Outcome ~ PRS + age + sex + pack-years), and Model 3 (Outcome ~ family history + PRS + age + sex + pack-years). B) COPDGene AA adjusted R^2 analyses. Abbreviations are as listed in caption for Table S7. The PRS was treated as a continuous variable.

A) COPDGene NHW

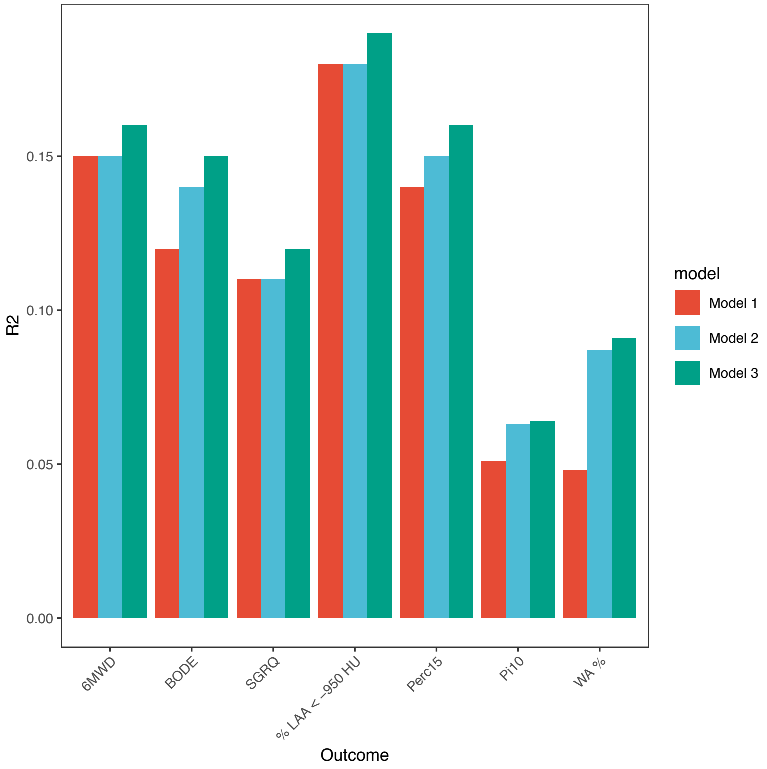

B) COPDGene AA

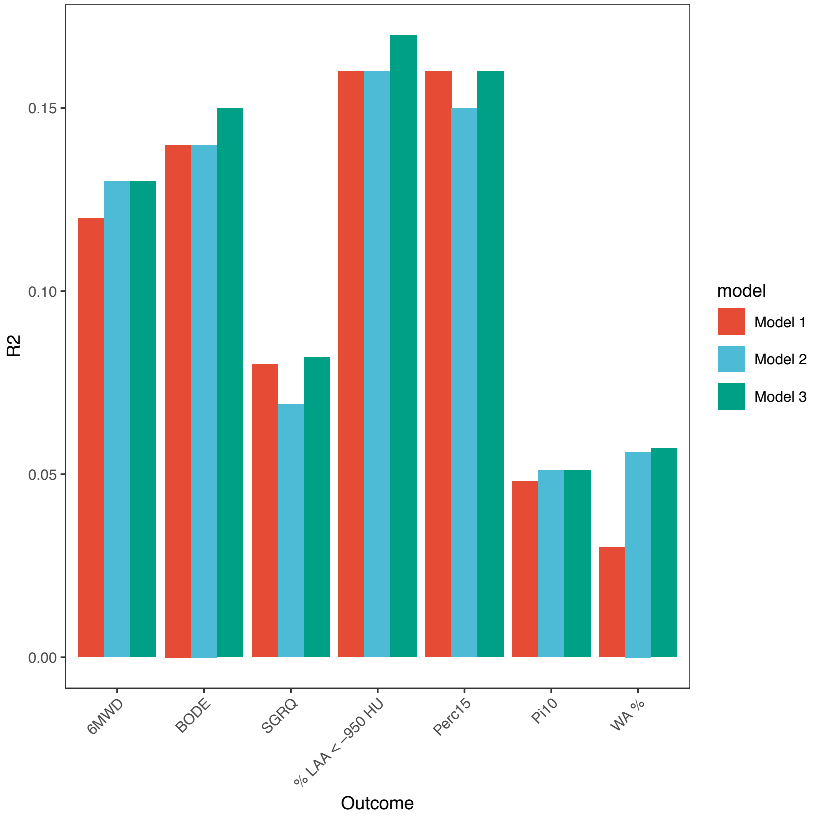

Figure S3: Predictive performance (AUC) of three logistic regression models for the discrimination of outcomes shown on the *x-axis* in the COPDGene study. The PRS was dichotomized (top versus bottom 2 tertiles). For each outcome, three models were trained: Model 1 (Outcome ~ family history + age + sex + pack-years), Model 2 (Outcome ~ PRS + age + sex + pack-years), and Model 3 (Outcome ~ family history + PRS + age + sex + pack-years). Abbreviations are as listed in caption for Table S7.
A: AUC analysis in COPDGene NHW

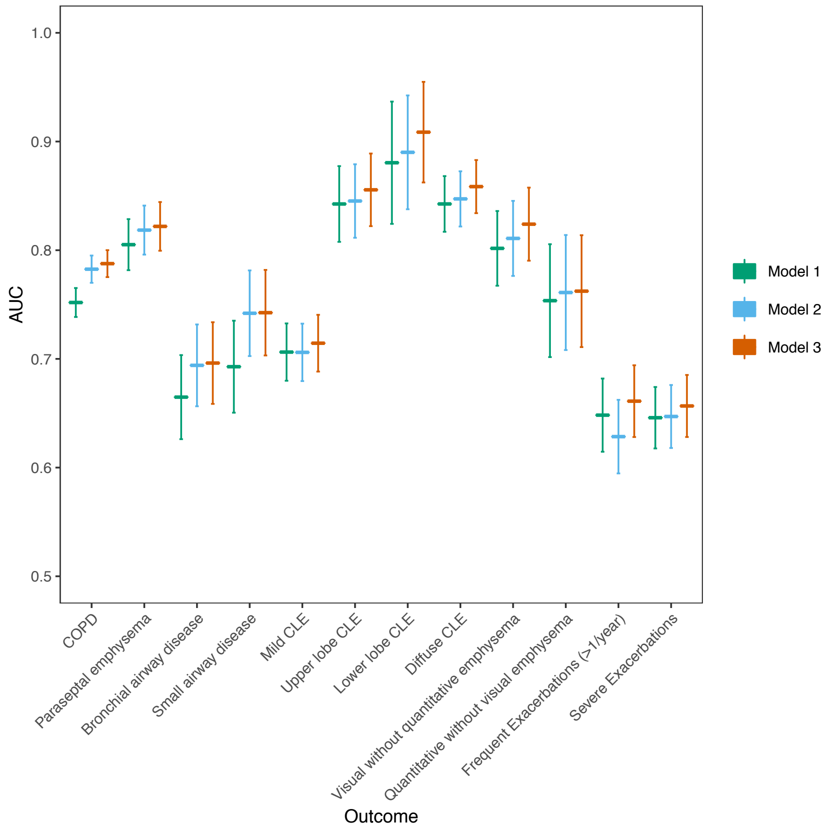

B: AUC analysis in COPDGene AA

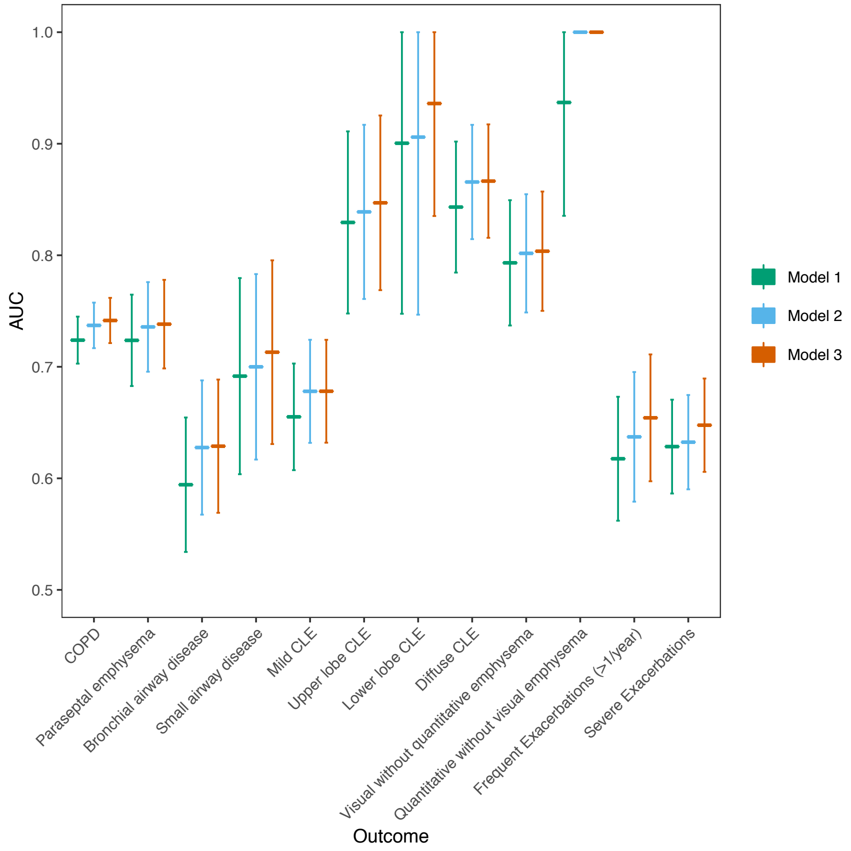

Figure S4: ECLIPSE AUC and R^2 with PRS treated as continuous variable.

A) Predictive performance (AUC) of three logistic regression models for the discrimination of outcomes shown on the *x-axis* in the ECLIPSE study. The PRS was analyzed as a continuous variable. For each outcome, three models were trained: Model 1 (Outcome ~ family history + age + sex + pack-years), Model 2 (Outcome ~ PRS + age + sex + pack-years), and Model 3 (Outcome ~ family history + PRS + age + sex + pack-years). Abbreviations are as listed in caption for Table S7.

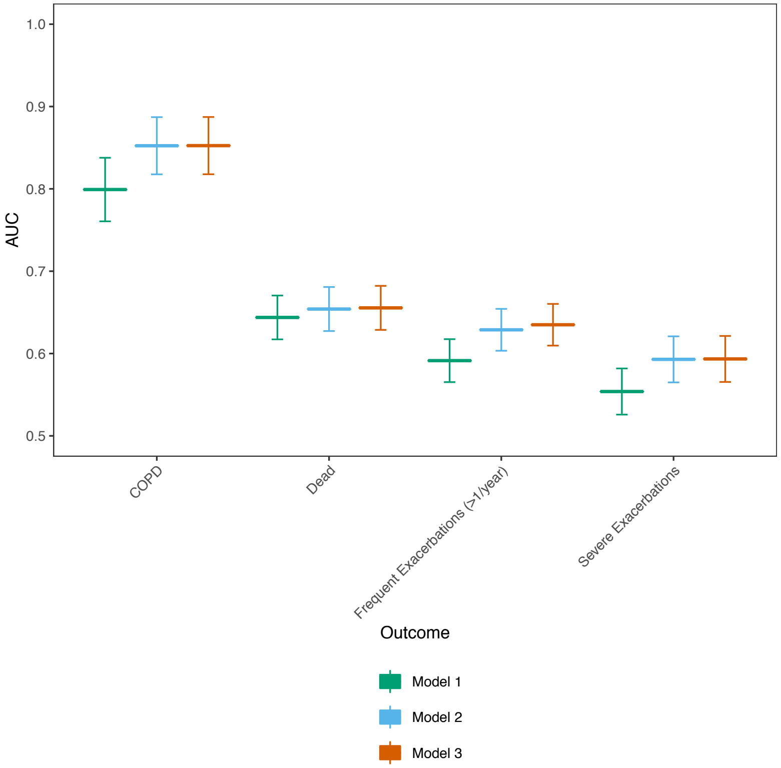

B) Predictive performance (R^2) of three linear regression models for outcomes shown on the *x-axis* in the ECLIPSE study. For each outcome, three models were trained: Model 1 (Outcome ~ family history + age + sex + pack-years), Model 2 (Outcome ~ PRS + age + sex + pack-years), and Model 3 (Outcome ~ family history + PRS + age + sex + pack-years). Abbreviations are as listed in caption for Table S7.

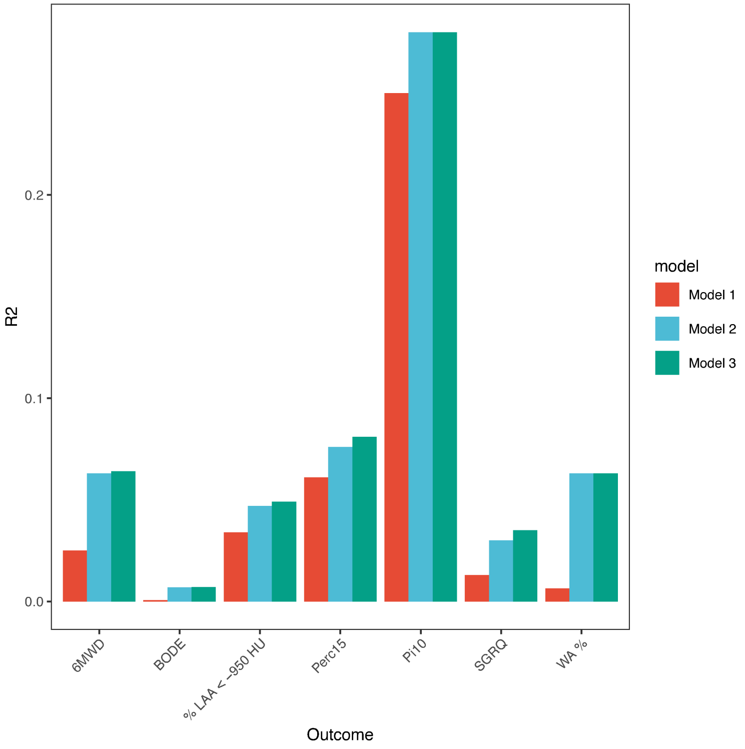

Figure S5: ECLIPSE AUC and R^2 with PRS dichotomized (top versus bottom 2 tertiles).

A) Predictive performance (AUC) of three logistic regression models for the discrimination of outcomes shown on the *x-axis* in the ECLIPSE study. For each outcome, three models were trained: Model 1 (Outcome ~ family history + age + sex + pack-years), Model 2 (Outcome ~ PRS + age + sex + pack-years), and Model 3 (Outcome ~ family history + PRS + age + sex + pack-years). "*" indicates that the p-value comparing model P-values comparing model AUCs were considered significant if less than Bonferroni-corrected level of significance (p < 0.05/4 = 0.013). Abbreviations are as listed in caption for Table S7.

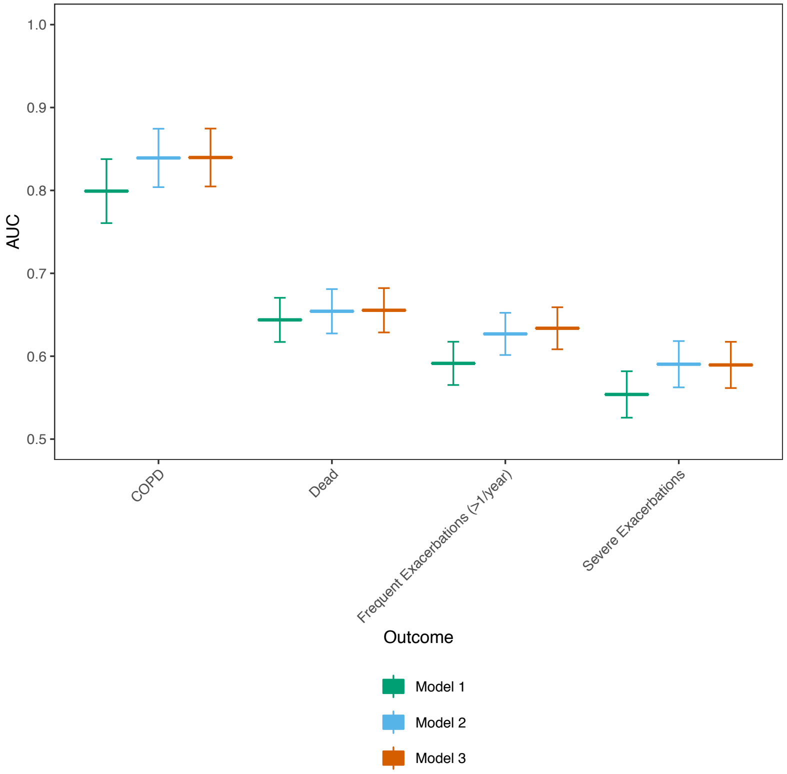

B) Predictive performance (R^2) of three linear regression models for outcomes shown on the *x-axis* in the ECLIPSE study. For each outcome, three models were trained: Model 1 (Outcome ~ family history + age + sex + pack-years), Model 2 (Outcome ~ PRS + age + sex + pack-years), and Model 3 (Outcome ~ family history + PRS + age + sex + pack-years). Abbreviations are as listed in caption for Table S7.

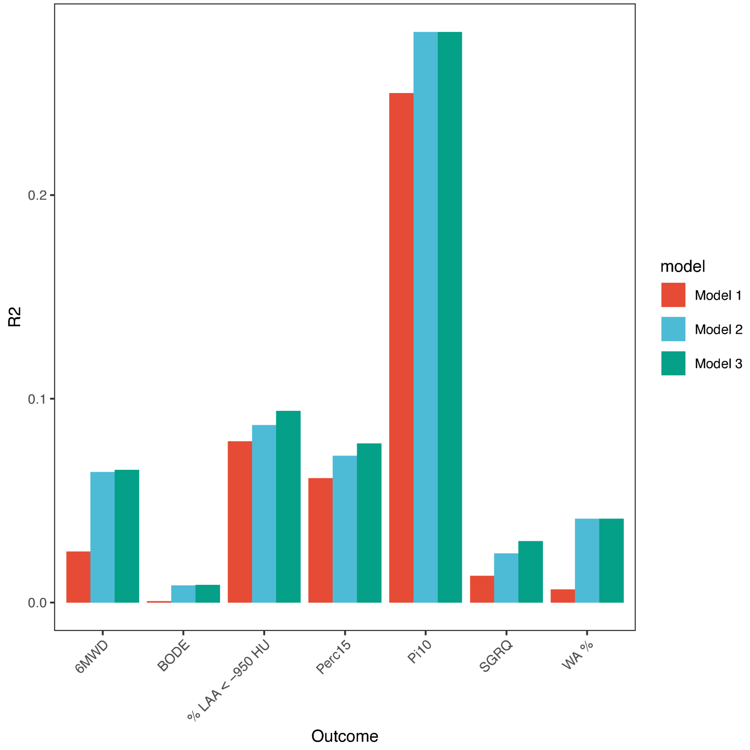

Moll M, *et al. BMJ Open Resp Res* 2020; 7:e000755. doi: 10.1136/bmjresp-2020-000755

Figure S6: Meta-analyses for linear outcomes with PRS treated as a continuous variable. COPDGene and ECLIPSE studies were meta-analyzed, and fixed effects beta coefficients with 95% confidence are shown for family history and PRS for each outcome. P-values were considered significant if less than Bonferroni-corrected level of significance (0.05/11 = 0.0045 (includes 4 binary outcomes)). Abbreviations are as listed in caption for Table S7.

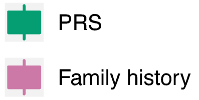

A) Clinical outcomes

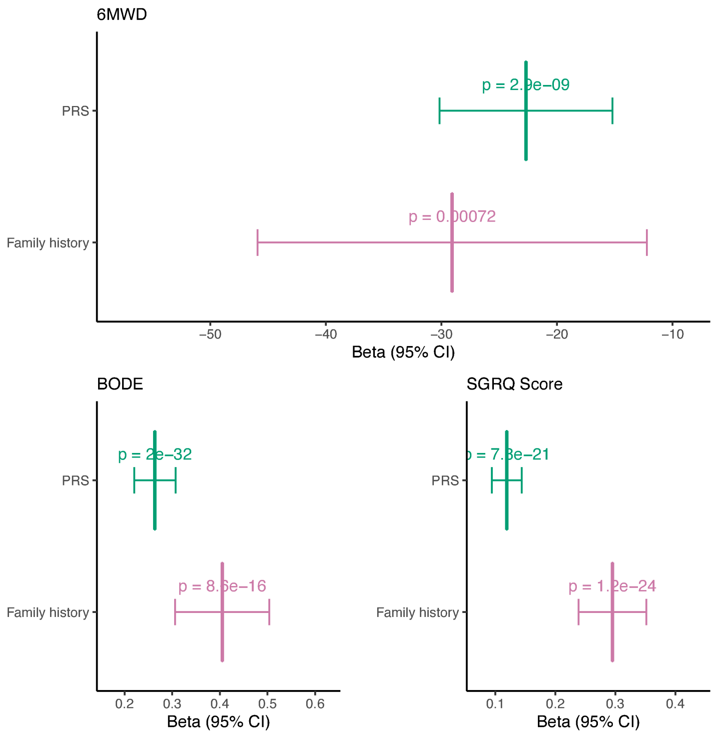

B) Imaging outcomes

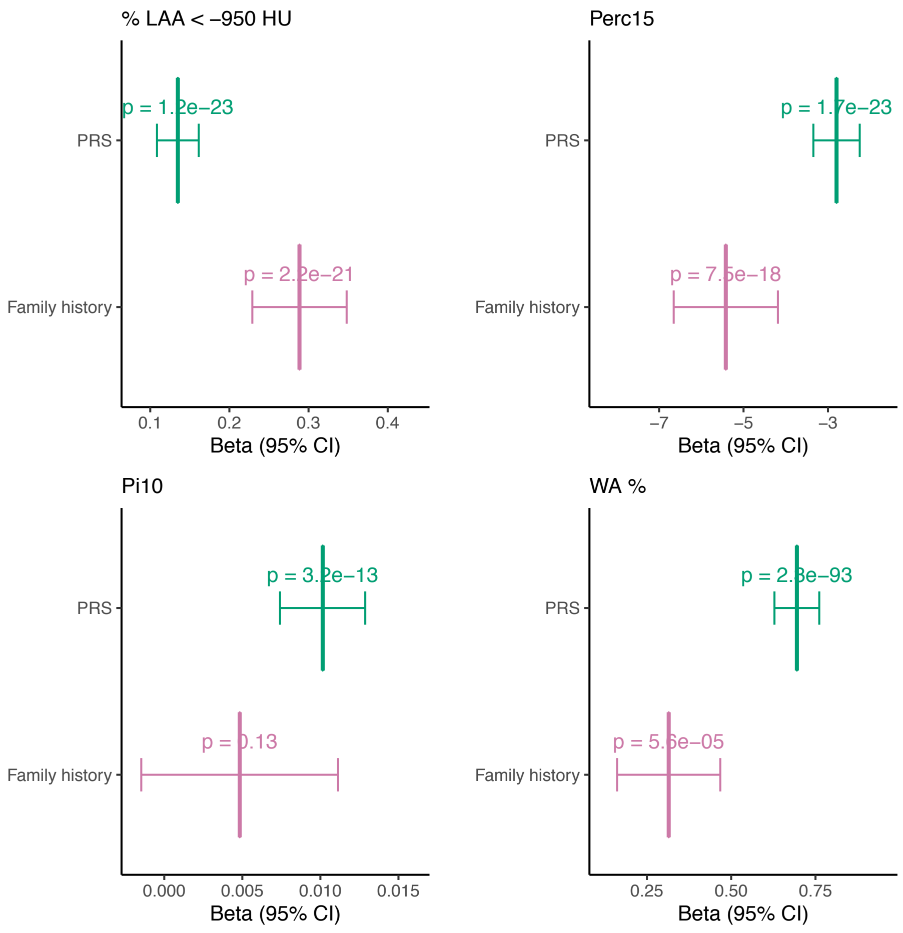

Figure S7: Meta-analyses of binary outcomes with a dichotomized PRS (top versus bottom 2 tertiles). COPDGene and ECLIPSE studies were meta-analyzed, and fixed effects odds ratios with 95% confidence are shown for family history and PRS for each outcome. Odds ratios for the PRS indicate the odds ratio for the listed outcome for every standard deviation increase in the PRS. P-values were considered significant if less than Bonferroni-corrected level of significance (0.05/11 = 0.0045 (includes 7 continuous outcomes).

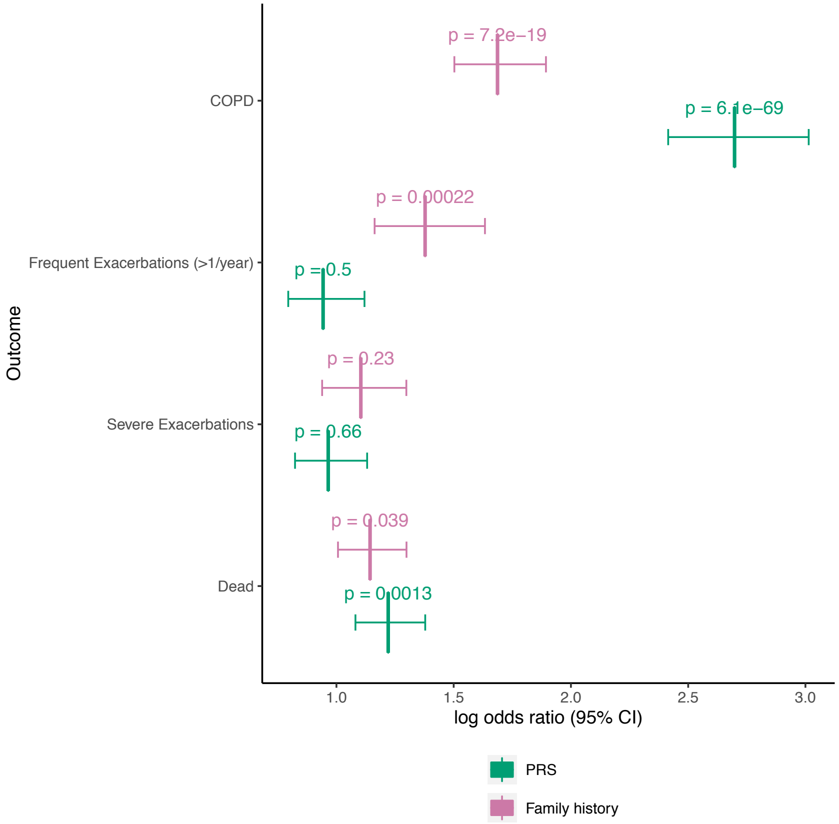

Moll M, *et al. BMJ Open Resp Res* 2020; 7:e000755. doi: 10.1136/bmjresp-2020-000755

Figure S8: Meta-analyses for linear outcomes with a dichotomized PRS (top versus bottom 2 tertiles). COPDGene and ECLIPSE studies were meta-analyzed, and fixed effects beta coefficients with 95% confidence are shown for family history and PRS for each outcome. P-values were considered significant if less than Bonferroni-corrected level of significance (0.05/11 = 0.0045 (includes 4 binary outcomes)). Abbreviations are as listed in caption for Table S7.

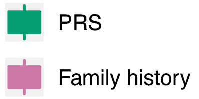

A) Clinical outcomes

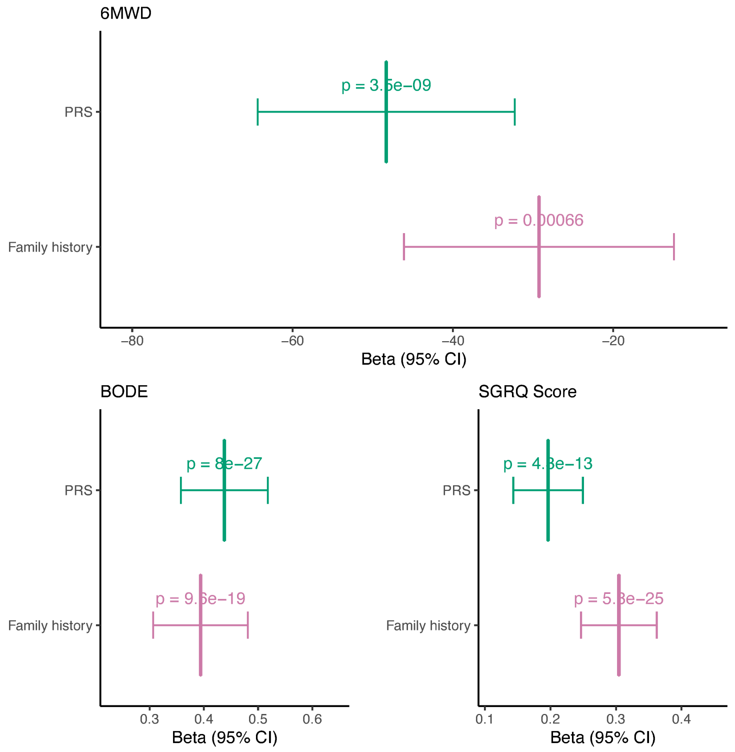

Moll M, *et al. BMJ Open Resp Res* 2020; 7:e000755. doi: 10.1136/bmjresp-2020-000755

B) Imaging outcomes

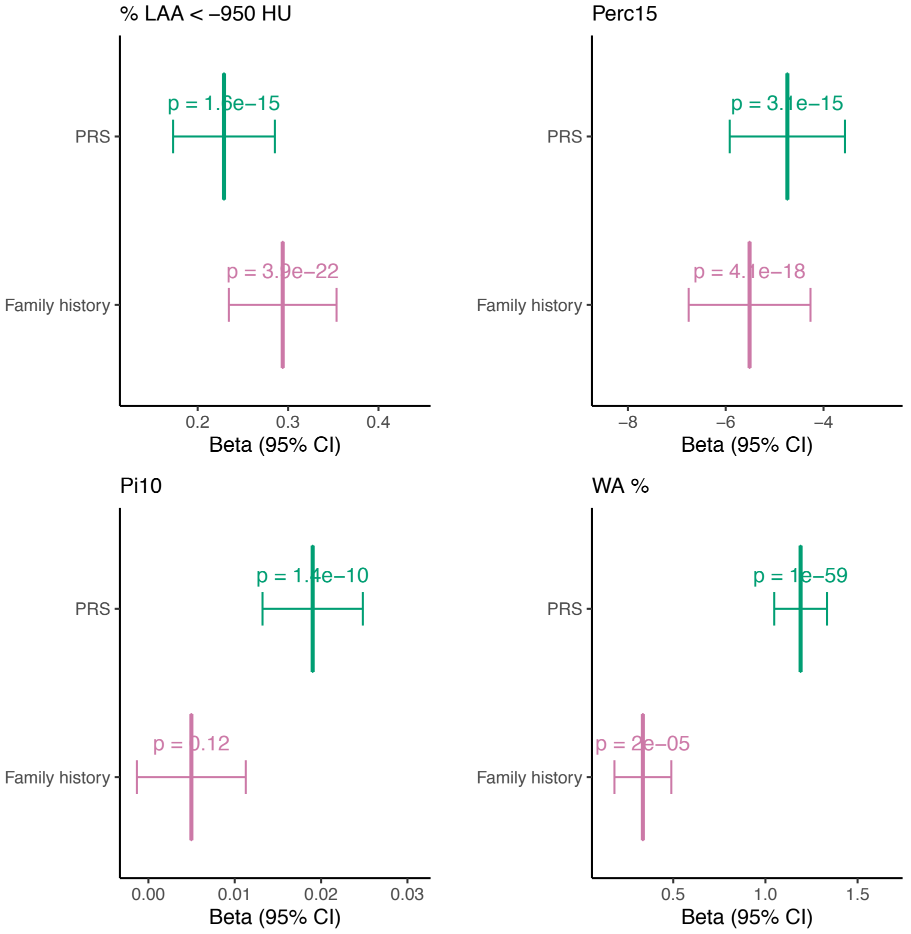

Moll M, *et al. BMJ Open Resp Res* 2020; 7:e000755. doi: 10.1136/bmjresp-2020-000755

Figure S9: Comparison of odd ratio for COPD for every standard deviation increase in the PRS in those with (*red*) and without (*blue*) a family history of COPD. 95% confidence bands are shaded in gray.
A) COPDGene NHW

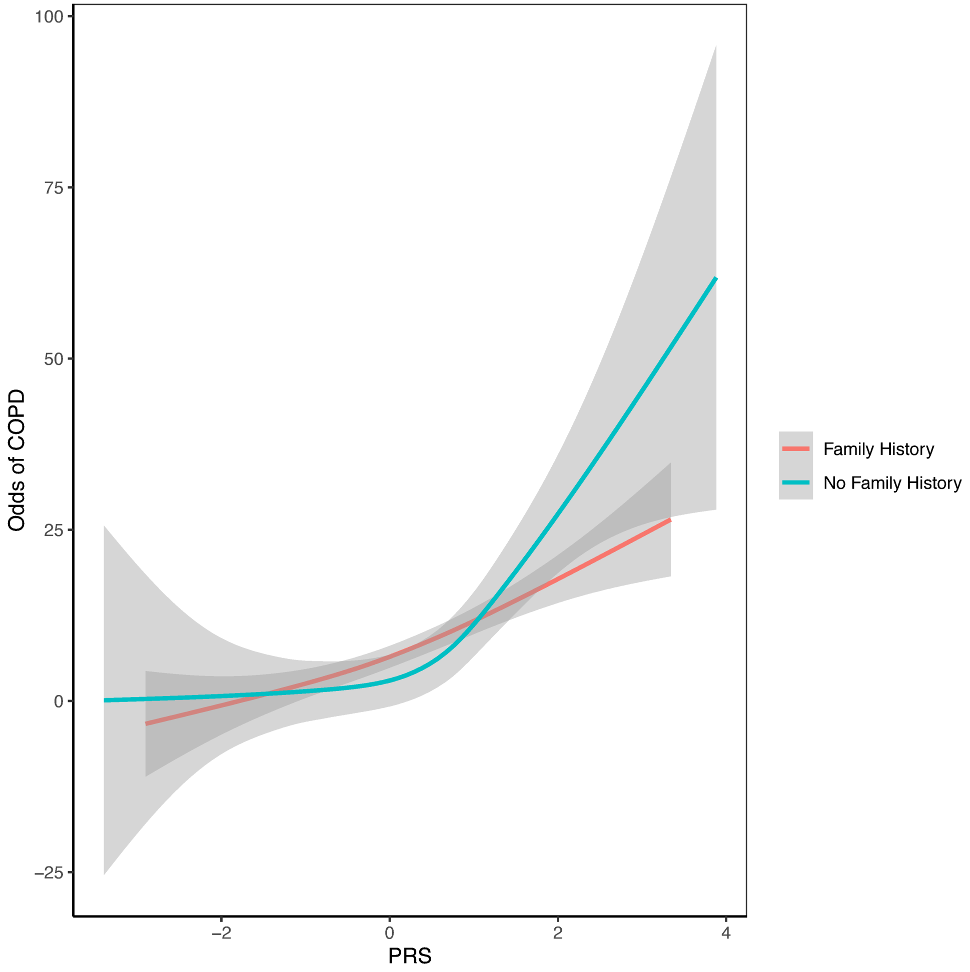

B) COPDGene AA

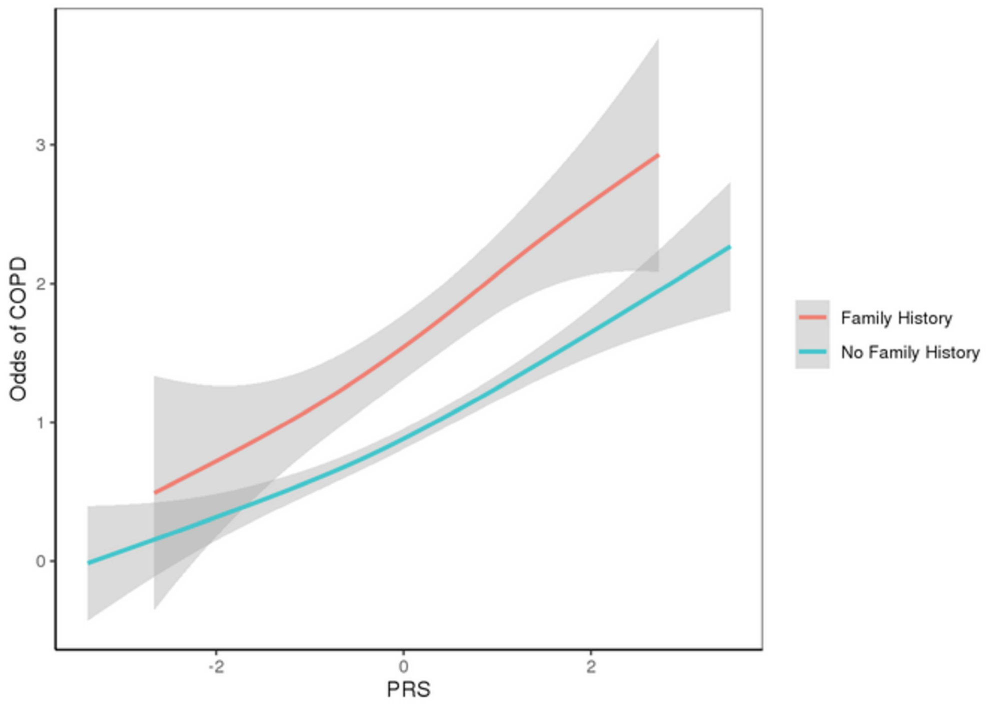

Moll M, et al. BMJ Open Resp Res 2020; 7:e000755. doi: 10.1136/bmjresp-2020-000755

Figure S10: Directed acyclic graph (DAG) showing the hypothesized natural effects model that was utilized for mediation analyses. Covariates include age, sex, pack-years of cigarette smoking, and principal components of genetic ancestry.

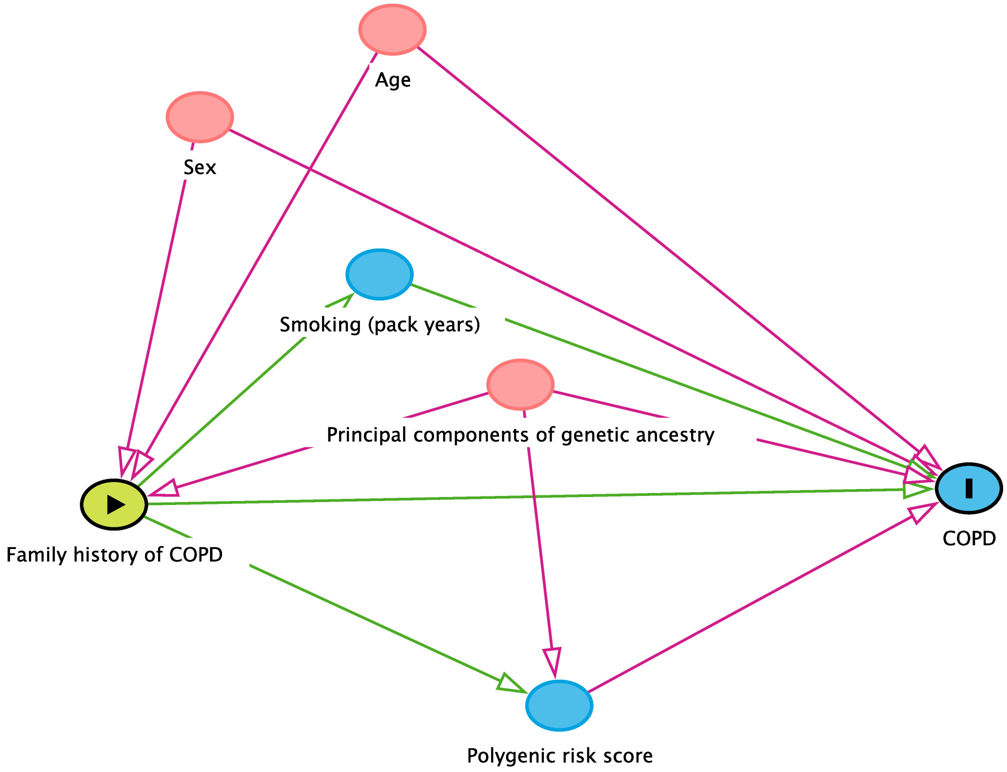

Moll M, *et al. BMJ Open Resp Res* 2020; 7:e000755. doi: 10.1136/bmjresp-2020-000755

References

1 Regan EA, Hokanson JE, Murphy JR, *et al.* Genetic Epidemiology of COPD (COPDGene) Study Design. *COPD J Chronic Obstr Pulm Dis* 2011; **7**: 32–43.
2 McCarthy S, Das S, Kretzschmar W, *et al.* A reference panel of 64,976 haplotypes for genotype imputation. *Nat Genet* 2016; **48**: 1279–83.
3 Vestbo J, Anderson W, Coxson HO, *et al.* Evaluation of COPD Longitudinally to Identify Predictive Surrogate End-points (ECLIPSE). *Eur Respir J* 2008; **31**: 869–73.
4 Celli BR, Cote CG, Marin JM, *et al.* The body-mass index, airflow obstruction, dyspnea, and exercise capacity index in chronic obstructive pulmonary disease. *N Engl J Med* 2004; **350**: 1005–12.
5 Steyerberg EW, Vickers AJ, Cook NR, *et al.* Assessing the performance of prediction models: a framework for traditional and novel measures. *Epidemiology* 2010; **21**: 128–38.
6 Leisman DE, Harhay MO, Lederer DJ, *et al.* Development and Reporting of Prediction Models: Guidance for Authors From Editors of Respiratory, Sleep, and Critical Care Journals. *Crit Care Med* 2020; **48**: 623–33.
